# A non-invasive secreted protein-based gene signature for prognostic stratification and tumor microenvironment assessment in gastric cancer

**DOI:** 10.7717/peerj.20517

**Published:** 2026-01-13

**Authors:** Qiuxia Liu, Haofeng Yin, Ziming Wang, Qianlong Shen, Jianguo Zhao, Xianhe Xie

**Affiliations:** 1 Department of Oncology, Molecular Oncology Research Institute, The First Affiliated Hospital, Fujian Medical University, Fuzhou, China; 2 Department of Oncology, National Regional Medical Center, Binhai Campus of The First Affiliated Hospital, Fujian Medical University, Fuzhou, China; 3 Department of Medical Oncology, Shaoxing People’s Hospital, Shaoxing Hospital of Zhejiang University, Shaoxing, China; 4 The First Affiliated Hospital, Shaoxing University, Shaoxing, China

**Keywords:** Gastric cancer, Secreted proteins, SERPINE1, Cancer associated fibroblasts, TME

## Abstract

**Background:**

Gastric cancer (GC) is a highly heterogeneous malignancy with poor prognosis. Current prognostic models for GC rely on invasive tissue-based high-throughput sequencing. Secreted proteins, detectable non-invasively and involved in tumor microenvironment (TME) remodeling, offer promising biomarkers. We aimed to develop a non-invasive prognostic signature based on secreted protein-coding genes (SPCGs) to stratify GC patients and predict TME characteristics.

**Methods:**

We obtained RNA sequencing data and clinical information from 375 GC and 32 paracancerous tissue samples from The Cancer Genome Atlas Stomach Adenocarcinoma (TCGA-STAD). Differentially expressed SPCGs were identified by intersecting differentially expressed genes with 731 Human Protein Atlas (HPA) secreted protein genes. An 8-SPCG signature was constructed using univariate Cox regression and least absolute shrinkage and selection operator (LASSO) regression analyses. The model’s predictive performance was validated through Kaplan–Meier survival curves, time-dependent receiver operating characteristic (ROC) analysis, and multivariable Cox regression. A nomogram integrating risk scores and clinical parameters was developed and validated using calibration curves. Functional annotation was conducted through Gene Ontology (GO), Kyoto Encyclopedia of Genes and Genomes (KEGG), and Gene Set Enrichment Analysis (GSEA). Tumor mutational burden (TMB) profiles and immune cell infiltration were compared between risk subgroups. The biological properties and clinical significance of SERPINE1 were validated through *in vitro* experiments and clinical data from our center.

**Results:**

An 8-SPCG signature (SERPINE1, C6, GRP, GCG, IL1F10, IGFBP1, ITIH2, and APOD) was identified and validated to predict overall survival in GC patients. The risk score derived from this signature was significantly associated with TME characteristics, including TME scores, immune cell infiltration, and immune checkpoint expression. High-risk patients exhibited an immunosuppressive microenvironment and lower TMB. Functional enrichment analysis indicated that the high-risk group was enriched in extracellular matrix-related pathways, while the low-risk group was associated with cellular metabolism and gene expression pathways. SERPINE1 was overexpressed in GC tissues, peripheral blood, and malignant effusions, and its high expression correlated with poor prognosis. *In vitro* experiments demonstrated that SERPINE1 promoted GC cell proliferation and invasion, and its expression was enhanced by cancer-associated fibroblasts (CAFs) through the EGF-ERBB signaling pathway.

**Conclusions:**

We established a non-invasive 8-SPCG signature that may serve as a potential predictor for GC prognosis and TME features. SERPINE1 was identified as a promising mediator linking GC progression to CAFs interactions, supporting its further investigation as a therapeutic target.

## Introduction

Despite a general trend of decreasing incidence and mortality rates, gastric cancer (GC) remains a highly prevalent malignant tumor and is the third leading cause of cancer-related mortality worldwide (Sung et al., 2021). This malignancy exhibits pronounced heterogeneity across epidemiological, genetic, histopathological, and behavioral dimensions, resulting in divergent clinical outcomes even among patients with comparable clinicopathological profiles. While recent advancements in surgical techniques and systemic therapies, including chemotherapy, targeted therapy, and immune checkpoint inhibitors, have improved treatment options, overall survival rates for GC patients remain unsatisfactory. Recurrence, distant metastasis, and drug resistance remain major obstacles to improving patient outcomes. Additionally, accumulating evidence suggests that tumor cells extensively interact with the tumor microenvironment (TME), influencing tumor initiation, proliferation, invasion, metastasis, and drug resistance (Balkwill, Capasso & Hagemann, 2012; Quail & Joyce, 2013; Ishimoto et al., 2014; Wang et al., 2017). Cancer-associated fibroblasts (CAFs), a key cellular component of the TME, interact with immune cells, extracellular matrix molecules, and various cytokines/chemokines/growth factors to collectively remodel the TME and drive GC initiation and progression (Ishimoto et al., 2014; Sun et al., 2022). These observations highlight the urgent need for robust biomarkers capable of stratifying GC patients based on TME characteristics to guide personalized therapeutic strategies.

Previous studies exploring biomarker models to predict prognosis in GC or TME features mostly selected key genes based on high-throughput sequencing (Gagan & Van Allen, 2015; Businello, Galuppini & Fassan, 2021; Su et al., 2024; Ren et al., 2021). However, this approach requires large quantities of high-quality tissue specimens, and obtaining tumor tissue is often labor-intensive and invasive. In contrast, secreted proteins, which serve as critical regulators of cell–cell signaling in the TME, present distinct advantages as non-invasive biomarkers (Balkwill, Capasso & Hagemann, 2012; Qin et al., 2023; Hoekstra et al., 2024). Their systemic detectability in plasma and serous effusions, coupled with roles in tumor-stroma crosstalk, positions them as promising candidates for clinical translation (Pavlou & Diamandis, 2010; May, 2009). While conventional serum biomarkers like carcinoembryonic antigen (CEA) and carbohydrate antigen (CA) 19-9 have demonstrated prognostic utility, their suboptimal sensitivity in early-stage disease and limited capacity to predict TME dynamics restrict broader clinical application (Shibata et al., 2022; Ucar et al., 2008; Chen et al., 2022a; Shimada et al., 2014).

To address the limitations of existing biomarker models based on high-throughput gene sequencing and single serum biomarkers, and to further investigate the biological significance of secreted proteins in GC, we examined the mRNA database of secreted proteins that can be tested in peripheral blood. We subsequently developed an eight-gene signature based on secreted protein-coding genes (SPCGs) and assessed its potential utility in predicting patient survival and TME characteristics. SERPINE1, a key component of this model, was further validated through multi-level analyses. Our findings indicated SERPINE1 overexpression in GC tissues, peripheral blood, and malignant effusions, correlating with advanced disease stages and aggressive histopathological features. Functional assays suggested potential roles for SERPINE1 in promoting proliferation, migration, and interaction with CAFs. Together, the findings from this study may provide a basis for a non-invasive approach to deciphering GC heterogeneity, potentially contributing to the future development of TME-informed biomarkers.

## Materials and Methods

### Data acquisition and differentially expressed gene screening

RNA sequencing profiles and clinical data for 375 GC tissues and 32 adjacent normal tissues were retrieved from The Cancer Genome Atlas Stomach Adenocarcinoma (TCGA-STAD; https://portal.gdc.cancer.gov/). An independent validation cohort (GSE15459, *n* = 200 GC cases) was obtained from the Gene Expression Omnibus (GEO; https://www.ncbi.nlm.nih.gov/geo/). To focus on blood-detectable secreted proteins, a list of 731 SPCGs was sourced from the Human Protein Atlas (HPA; https://www.proteinatlas.org/humanproteome/blood+protein/secreted+to+blood) (Chen et al., 2022c). Differential expression analysis between tumor and normal tissues was performed using the “limma” R package, with significance thresholds set at |log2 fold change| > 1 and false discovery rate (FDR)-adjusted *p* < 0.05. SPCGs overlapping with DEGs were visualized *via* Venn diagram.

### Prognostic signature construction

Univariate Cox proportional hazard model analysis was conducted on each SPCGs to identify genes significantly associated with overall survival (OS) in the training dataset, using a log-rank *P*-value of less than 0.05 as the threshold. This analysis identified 57 genes with prognostic significance for OS prediction. Subsequently, the least absolute shrinkage and selection operator (LASSO) regression method was employed to reduce the number of genes, using the “glmnet” and “survival” packages. The SPCGs risk signature for calculating individual risk scores was developed based on nonzero coefficients from the LASSO regression analysis. The formula was established as follows: risk score = Σ (coefi×expi), where coefi represents the coefficient and expi denotes the expression level of each gene.

### Model validation and nomogram development

Patients were stratified into high-risk and low-risk groups based on their median risk scores derived from the training set. Differences in OS time between the groups were evaluated using log-rank tests and Cox regression analyses. Kaplan–Meier survival analysis was employed to compare survival rates between the two groups, using the “survival” and “survminer” packages in R software. The receiver operating characteristic (ROC) curve was used to evaluate the model’s predictive accuracy for 1-, 2-, 3-, and 5-year OS survival, using the “survival”, “survminer”, and “timeROC” packages. The predictive value of the prognostic model was further validated using the GEO database as a validation set, following the same methodologies. Age, sex, grade, TNM stage, and risk score were incorporated into both univariate and multivariate Cox regression analyses using the “survival” package to identify independent variables associated with OS. A nomogram based on the 8-SPCG signature was developed using the R packages “rms” and “regplot”, incorporating variables such as risk score, age, gender, grade, and stage. A calibration plot was used to validate the predictive accuracy of the nomogram for 1-, 3-, and 5-year OS in GC patients.

### Functional enrichment analysis

Gene set enrichment analysis (GSEA) was performed using the “clusterProfiler” R package, based on the Kyoto Encyclopedia of Genes and Genomes (KEGG) pathway and Gene Ontology (GO) analyses. Additionally, gene set variation analysis (GSVA) was conducted using the “GSVA” R package to explore the relationship between the expression of SPCGs, risk score, and KEGG pathways.

### TMB and immune microenvironment analysis

The TME landscape was comprehensively analyzed, focusing on tumor mutation burden (TMB), immune cell infiltration, immune checkpoint expression, and immune-associated scores within the TCGA-STAD database. TMB was assessed using the “maftools” package. The ESTIMATE algorithm was employed to calculate stromal, immune, and ESTIMATE scores using the “estimate” package. The composition of tumor-infiltrating immune cells within the TME was elucidated using multiple computational tools, including XCELL, TIMER, QUANTISEQ, MCPcounter, EPIC, CIBERSORT-ABS, and the CIBERSORT algorithm, along with the LM22 signature matrix file downloaded from (http://cibersort.stanford.edu/). Spearman’s correlation analysis was carried out to examine the relationship between the risk score, signature genes, immune cell composition, and immune checkpoint gene expression. The immunophenoscore (IPS) was used to predict the response to immune checkpoint inhibitors in high-risk and low-risk groups, based on the positive or negative status of programmed cell death ligand 1 (PD-L1) and cytotoxic T-lymphocyte-associated protein 4 (CTLA-4). IPS data were sourced from the Cancer Immunome Database (TCIA) (https://tcia.at/home).

### Clinical samples

Clinical samples were obtained from Shaoxing People’s Hospital. This study utilized 56 formalin-fixed, paraffin-embedded GC tissue samples (52 for SERPINE1 and 47 for α-smooth muscle actin (α-SMA) staining), peripheral blood samples from GC patients, and effusion fluids. Additionally, fresh GC and paired adjacent non-tumorous tissues were collected during surgery for transcriptome sequencing, western blot and primary fibroblast isolation. The study protocol was approved by the Hospital’s Academic Ethics Committee (Approval No: 2023 Scientific research project 104-Y-01). Due to the retrospective data analysis and use of leftover clinical specimens, informed consent for collecting clinical samples and corresponding clinical data was waived. However, informed consent for use of patient biospecimens and health information was still obtained from all patients providing fresh tissues.

### Cell lines and culture

Normal gastric epithelial cells (GES-1) and three human GC cell lines (AGS, HGC27, and MKN7) were used. AGS and MKN7 cell lines were obtained from Procell Life Science & Technology Co., Ltd (Wuhan, China), while the GES-1 and HGC27 cell lines were sourced from Cellverse Co., Ltd (Shanghai, China). All cell lines had the Short Tandem Repeat (STR) authentication. AGS and HGC27 cells were cultured in Dulbecco’s Modified Eagle Medium (DMEM) supplemented with 10% fetal bovine serum (FBS), whereas GES-1 and MKN7 cells were maintained in Roswell Park Memorial Institute (RPMI) 1640 medium with 10% FBS. All cells were incubated at 37 °C in a humidified atmosphere with 5% CO_2_.

### Isolation and cultivation of CAFs and NFs

CAFs and NFs were isolated from surgically resected gastric cancer tissues and adjacent non-cancerous tissues, respectively. The tissues were minced and digested with 0.15% collagenase type I for 4–6 h. The resulting cell suspension was filtered and cultured in DMEM containing 20% FBS. The cells were further isolated and purified through differential enzymatic digestion using Accutase cell detachment solution (Gibco, Billings, MT, USA).

### GC-CAF Co-culture system

An indirect co-culture was established using 0.4-µm pore-size Transwells. CAFs were plated in the upper chamber and AGS or HGC27 cells (1.5 × 10^5^) in the lower chamber. Cells were co-cultured in complete DMEM for 48 h before collection for analysis.

### Establishment and validation of stable SERPINE1 knockdown and overexpression cell lines

Lentiviral vectors from Sangon Biotech (Shanghai, China) were used to establish stable SERPINE1 knockdown and overexpression cell lines. Transduction efficiency was verified by reverse transcription quantitative polymerase chain reaction (RT-qPCR) and western blot. The functional impact was then assessed using plate colony formation and Transwell assays to evaluate clonogenic and migratory capacities, respectively.

### Plate colony formation assay and transwell assay

Cell clonogenicity was assessed by plating 400 cells per well in 6-well plates for 2 weeks. Resulting colonies were fixed with 4% formaldehyde, stained with 0.2% crystal violet, and quantified using an Enzyme-linked Spot Image Automatic Analyzer (CTL, USA). Cell migration was evaluated using 24-well Transwell chambers (Corning, NY, USA). GC cells (5 × 10^4^ for AGS/HGC27; 1 × 10^5^ for MKN7) in serum-free medium were seeded into the upper chamber, with complete medium as a chemoattractant in the lower chamber. After 24 h, migrated cells on the membrane were fixed, stained, and quantified. To assess CAF-induced migration, 1 × 10^5^ CAFs were plated in the lower chamber.

### mRNA sequencing and RT-qPCR

mRNA sequencing was performed on three pairs of fresh GC and adjacent normal tissues by Sangon Biotech using the MGISEQ-T7 platform.

For RT-qPCR, total RNA was extracted from cells, reverse transcribed into cDNA, and amplified using SYBR Green Master Mix on a QuantStudio 5 system. Gene expression was normalized to GAPDH and calculated using the ΔΔCt method. The primer sequences for SERPINE1 were as follows: forward primer, ACTGCCCTCTATTCAACGGC; reverse primer, GGGCGTGGTGAACTCAGTAT.

### Western blot analysis

Proteins were separated by sodium dodecyl sulfate-polyacrylamide gel electrophoresis (SDS-PAGE), transferred to polyvinylidene fluoride (PVDF) membranes, and blocked with 5% non-fat milk. Membranes were incubated overnight at 4 °C with primary antibodies against SERPINE1 (1:1000, Abcam, Cambridge, UK) and GAPDH (1:3000, ZENBIO, China), followed by a 2-hour incubation with a goat anti-rabbit secondary antibody (1:100,000, HUABIO, China) at room temperature. Signals were detected using an ECL kit (Beyotime, China) on a Bio-Rad ChemiDoc system. The original western blot images can be found in Fig. S1.

### Immunohistochemistry assay

IHC was performed on paraffin-embedded sections using standard protocols. Sections were incubated with primary antibodies against SERPINE1 (1:800, Abcam, Cambridge, UK) and α-SMA (1:800, Abcam, Cambridge, UK) overnight at 4 °C, followed by a secondary antibody (DAKO, Germany) and 3,3′-diaminobenzidine (DAB) visualization. Staining intensity was quantified using ImageJ by calculating the average optical density (AOD = Integrated Optical Density/Area) from five random fields per section.

### Enzyme-linked immunosorbent assay (ELISA)

SERPINE1 levels in peripheral blood, pleural/peritoneal fluids, and culture supernatants from cell lines/fibroblasts were quantified using Enzyme-linked immunosorbent assay (ELISA) kits from MultiSciences (Hangzhou, China) according to the manufacturer’s instructions. All fluid samples were centrifuged prior to analysis.

### Immunofluorescence staining

CAFs and NFs were plated on coverslips, fixed with 4% paraformaldehyde, and permeabilized with 0.1% Triton X-100. After blocking with 5% bovine serum albumin (BSA), cells were incubated with an α-SMA primary antibody (1:400, Abcam, Cambridge, UK) overnight at 4 °C, followed by an Alexa Fluor 488-conjugated secondary antibody (1:400, Solarbio, Beijing, China) for 1 h at room temperature. Nuclei were counterstained with 4′,6-diamidino-2-phenylindole (DAPI), and images were captured using a confocal microscope.

### Statistical analysis

Data from clinical and cellular experiments are expressed as mean ± standard deviation (SD) and were analyzed using GraphPad Prism 10 software (San Diego, CA, USA). T test or Wilcoxon-test were used for comparisons between two groups, while one-way ANOVA was used for comparisons among multiple groups. The relationship between the IHC score of SERPINE1 and α-SMA was assessed using Spearman’s correlation analysis, considering the non-normal distribution of the variables. Outliers were determined by the box plot method or Grubbs test with an α-value ≤ 0.05 (3≥ n ≤30). All data were included and outliers were not excluded. Statistical significance was defined as a *p*-value below 0.05, with significance levels indicated as: * for *p* < 0.05, ** for *p* < 0.01, and *** for *p* < 0.001.

## Results

### Identification of an 8-SPCG signature

**Figure 1:**
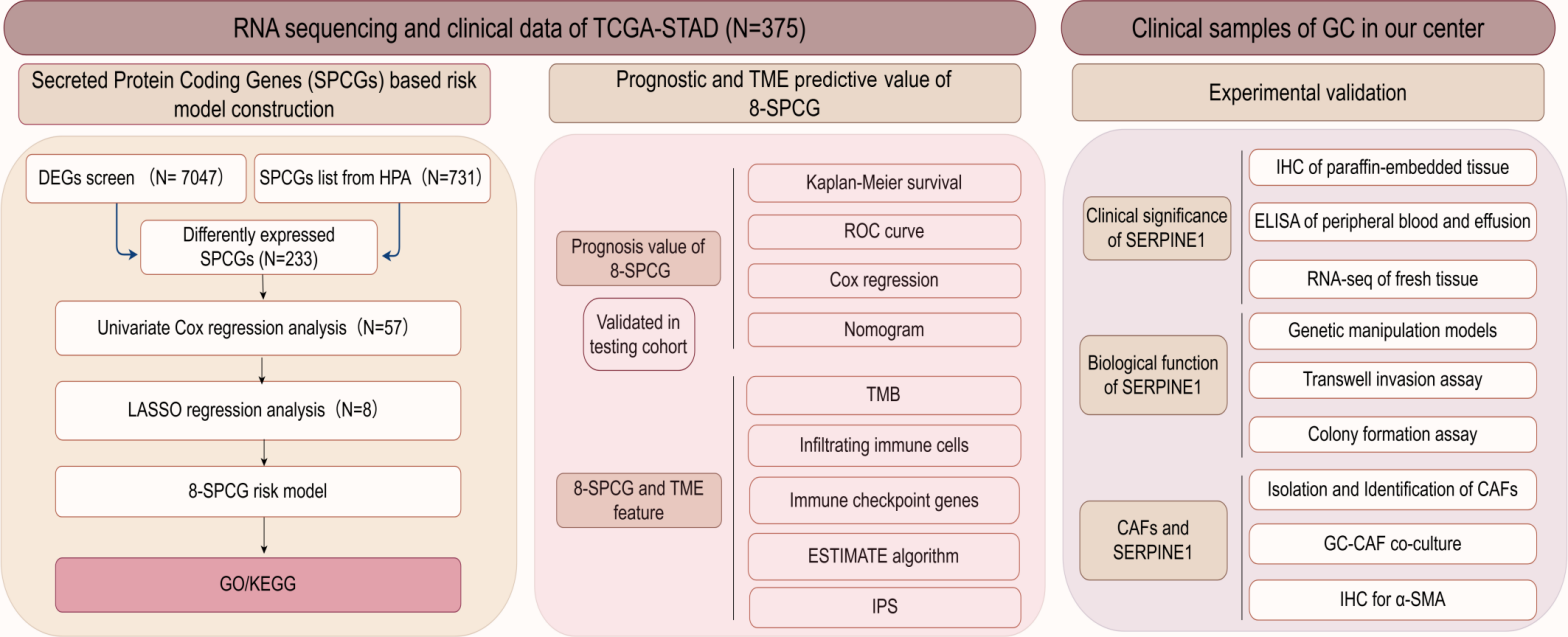
The flow diagram of this study.

**Figure 2:**
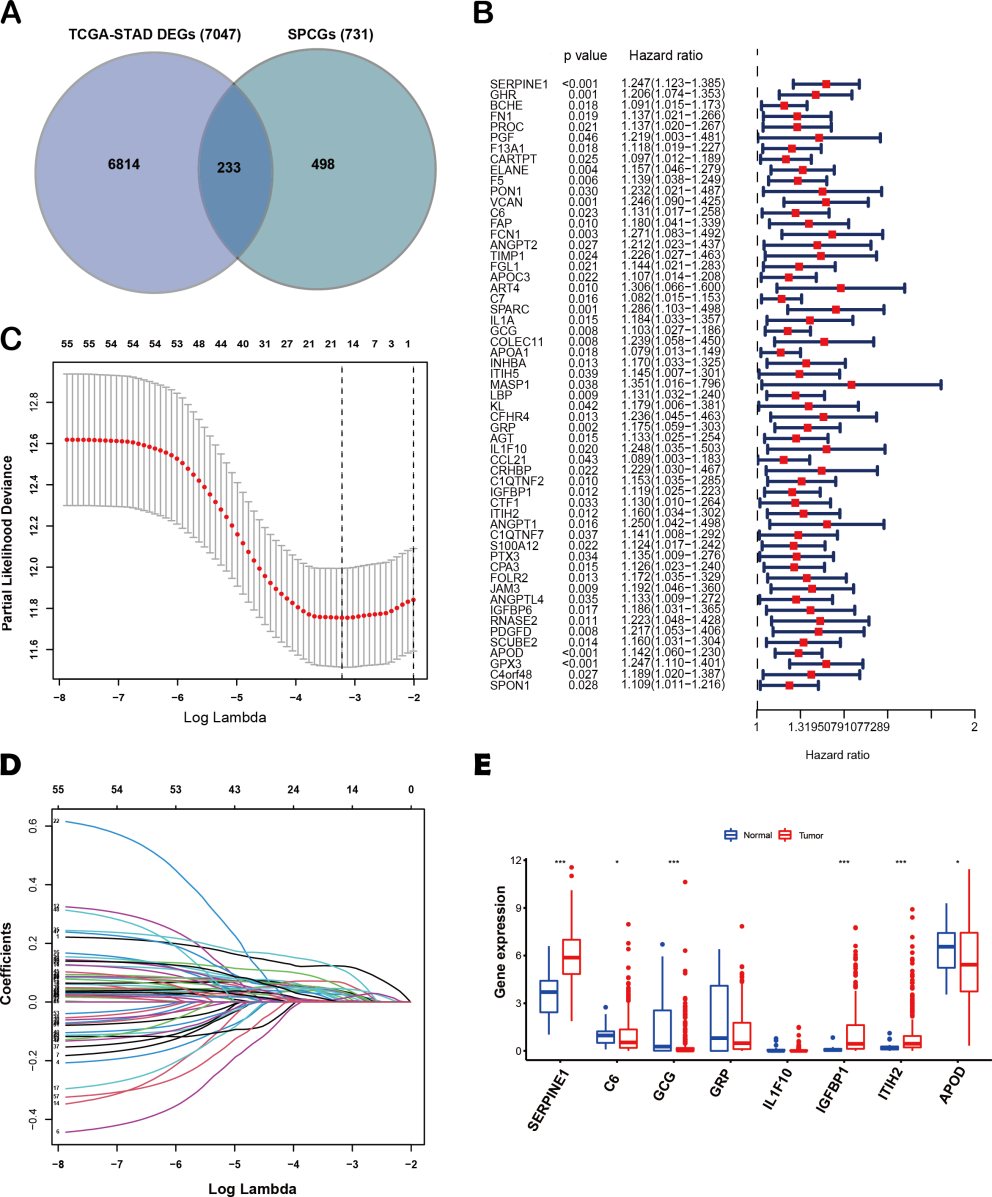
Identification of an 8-SPCG signature. (A) A Venn diagram illustrating the identification of 233 differentially expressed SPCGs, derived from the intersection of DEGs from TCGA-STAD and SPCGs from the HPA. (B) A forest plot depicting the prognostic significance of 57 SPCGs identified through univariate Cox regression analysis. (C) LASSO regression curve analysis of the 57 SPCGs. (D) Distribution of coefficients obtained from the LASSO regression. (E) Comparative expression analysis of the eight SPCGs between GC tumor tissues and adjacent normal tissues. Asterisks indicate statistical significance: *P < 0.05, ***P < 0.001.

The flow diagram of this study is shown in Fig. 1. A list of 233 differentially expressed SPCGs was compiled by intersecting the DEGs from the TCGA-STAD dataset with those encoding secreted proteins in the HPA (Fig. 2A). Among them, 57 SPCGs were found to be significantly associated with OS, as determined *via* univariate Cox regression analysis (Fig. 2B). Subsequent LASSO regression analysis identified an 8-gene signature comprising SERPINE1, C6, GRP, GCG, IL1F10, IGFBP1, ITIH2, and APOD (Figs. 2C, 2D). Analysis of differential expression between GC tumor tissues and normal tissues revealed upregulated SERPINE1, IL1F10, IGFBP1, and ITIH2, whereas C6, GCG, GRP, and APOD were downregulated (Fig. 2E). In summary, building upon data from the TCGA and HPA databases, an 8-SPCG signature was hereby identified through univariate Cox regression and LASSO regression analyses.

### Construction of an 8-SPCG prognostic risk model

**Figure 3:**
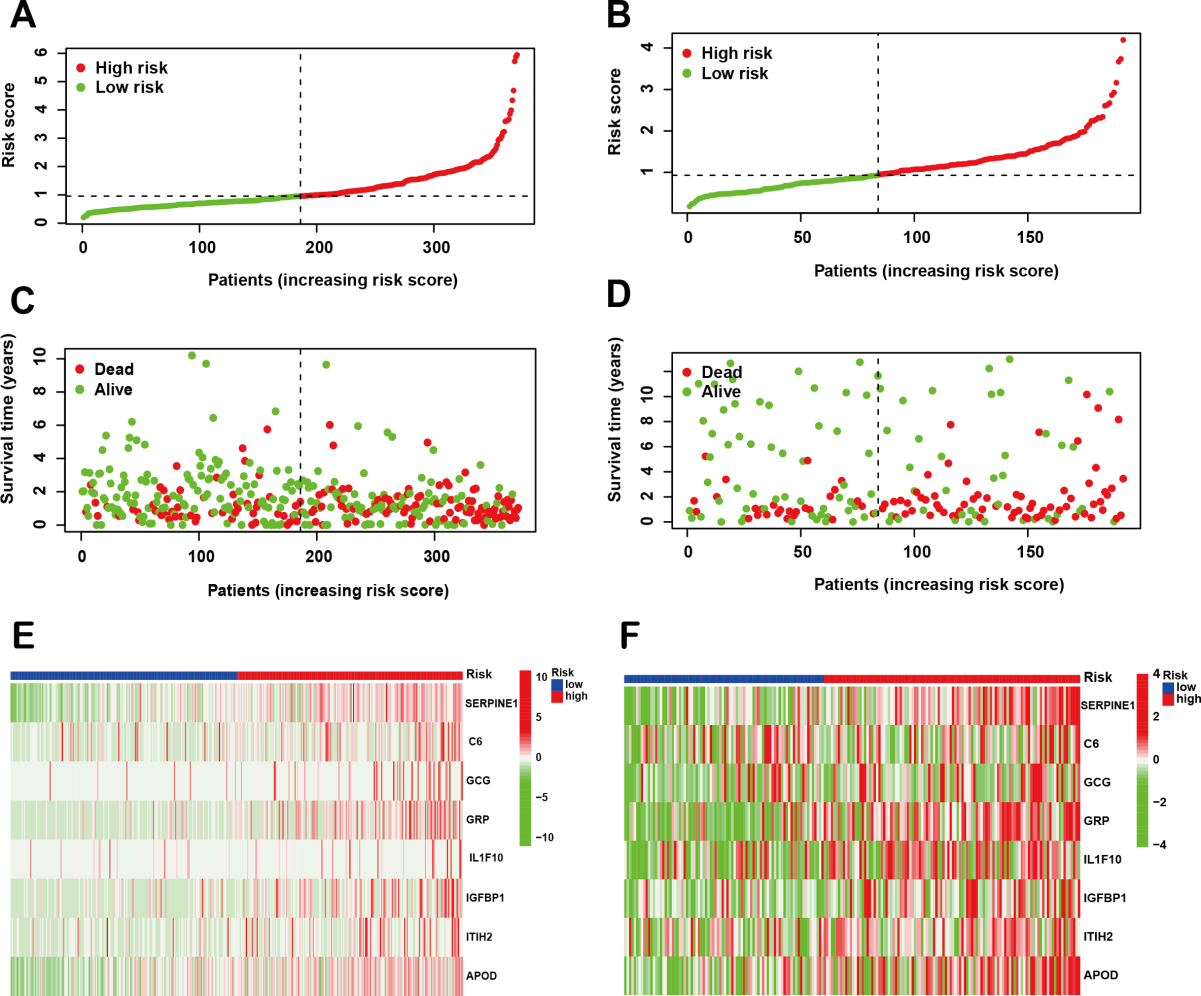
Construction of a prognostic risk model. (A, B) Risk score distribution in the TCGA training cohort (A) and GEO validation cohort (B). (C, D) Survival status with corresponding risk scores in the training (C) and validation sets (D). (E, F) Heat maps showing signature gene expression levels in the training (E) and validation groups (F).

Following the identification of the SPCG signature, we employed LASSO Cox regression analysis to establish a prognostic risk model using the TCGA-STAD cohort as the training set. This model was subsequently validated through independent analysis of the GEO database. Risk scores for individual patients were computed by integrating the expression profiles of the 8-SPCG with their respective regression coefficients derived from the LASSO analysis. Using the median risk score as the stratification threshold, GC patients were effectively stratified into distinct high-risk and low-risk subgroups. Visual representation of risk score distributions revealed clear bimodal patterns in both the training cohort (Fig. 3A) and validation cohort (Fig. 3B). Survival analysis demonstrated significant divergence between risk groups, with corresponding patient survival status and risk score gradients illustrated through scatter plots in the training set (Fig. 3C) and validation set (Fig. 3D). Comparative expression profiling *via* heatmap visualization confirmed differential expression patterns of all 8-SPCG between risk subgroups, with consistent patterns observed in both training (Fig. 3E) and validation cohorts (Fig. 3F). Collectively, results from the training and validation cohorts provide preliminary support for the ability of the 8-SPCG-derived risk model to distinguish patient outcomes, suggesting its exploratory value for prognostic assessment in GC.

### Validation the predictive value of 8-SPCG prognostic model

**Figure 4:**
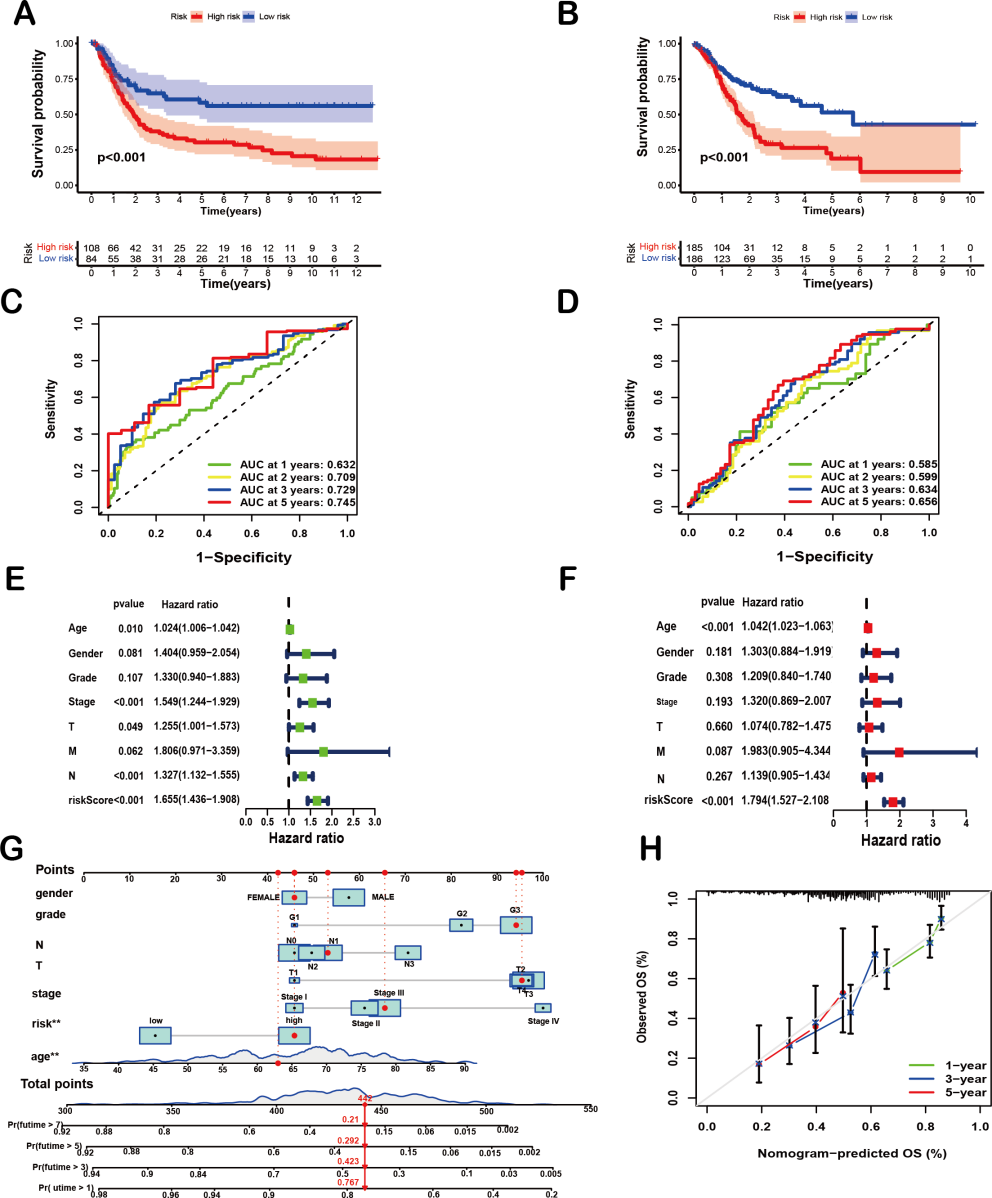
Prognostic and predictive value of the 8-SPCG signature. (A, B) Kaplan–Meier curves of overall survival time between high-risk and low-risk groups in training set (A) and validation set (B). (C,D) Receiver Operating Characteristic (ROC) analysis of the signature within the TCGA (C) and GEO databases (D). (E, F) Univariate Cox regression (E) and multivariate Cox regression analyses (F) for overall survival in the TCGA-STAD database. (G, H) Nomogram for survival prediction (G) and calibration curves of the nomogram (H) in the TCGA-STAD database.

To comprehensively evaluate the prognostic efficacy of the 8-SPCG signature, Kaplan–Meier survival analysis was performed. The resulting curves revealed a significant divergence in OS between high-risk and low-risk groups in both the training and validation cohorts. Specifically, in the TCGA-STAD training set (Fig. 4A), patients assigned to the high-risk group exhibited a markedly shorter OS compared to their low-risk counterparts (*p* < 0.001), a trend that was consistently replicated in the GEO validation set (Fig. 4B, *p* < 0.001). These findings strongly underscore the discriminatory power of the 8-SPCG-based risk model in stratifying patients with varying survival outcomes. Subsequently, ROC curve analysis was conducted to quantitatively assess the sensitivity and specificity of the prognostic model. Using data from the TCGA database, the time-dependent ROC curves demonstrated favorable discriminatory performance, with area under the curve (AUC) values of 0.632 for 1-year survival, 0.709 for 2-year survival, 0.729 for 3-year survival, and 0.745 for 5-year survival (Fig. 4C). These results were further validated in the GEO dataset (Fig. 4D), reinforcing the robustness of the model’s predictive capabilities across independent cohorts. To identify independent prognostic factors associated with OS, univariate and multivariate Cox regression analyses were carried out, incorporating the risk score and a comprehensive set of clinicopathological variables, including age, gender, tumor grade, T stage, N stage, M stage, and tumor stage. Univariate Cox regression analysis (Fig. 4E) initially screened potential predictors, while multivariate Cox regression analysis (Fig. 4F) adjusted for confounding factors to determine independent prognostic indicators. The results of these analyses unequivocally identified age and the 8-SPCG-derived risk score as independent predictors of OS in GC patients. Leveraging these findings, a nomogram was constructed, integrating the selected clinicopathological parameters from the regression analyses to facilitate individualized OS prediction for GC patients (Fig. 4G). The nomogram’s predictive accuracy was further evaluated using a calibration plot, which demonstrated excellent agreement between the predicted and observed survival probabilities at 1, 3, and 5 years (Fig. 4H). Therefore, results from multiple analytical approaches collectively suggest that the 8-SPCG signature may hold predictive value, supporting its further investigation as a potential tool for clinical decision-making and patient management in GC.

**Figure 5:**
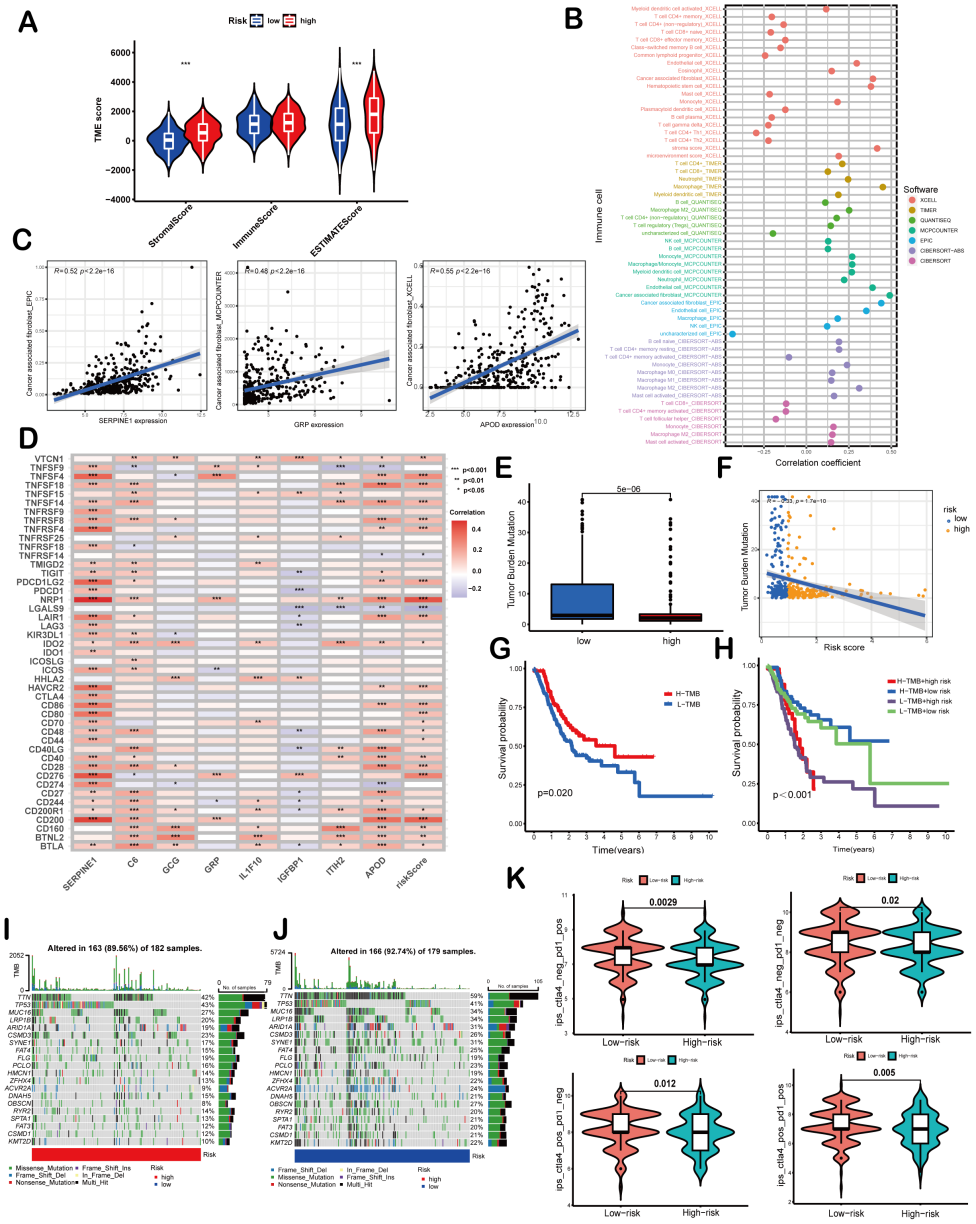
Correlation of the risk score with TMB and immune landscape of GC. (A) Differences in TME scores between high-risk and low-risk groups were assessed using the ESTIMATE algorithm. (B) An analysis of immune cell components revealed correlations between immune cells and risk scores. (C) Scatter plots demonstrated a positive relationship between SERPINE1, APOD, and GRP with cancer-associated fibroblasts, individually. (D) The association between risk scores and signature genes with immune checkpoints was evaluated using Spearman correlation analysis. (E) A comparison of TMB was conducted between high-risk and low-risk groups. (F) TMB was found to be negatively correlated with risk scores. (G, H) Kaplan–Meier curves illustrated the prognostic value of TMB (G) and the combination of TMB and risk scores (H). (I, J) Waterfall diagrams depicted the top 20 driver genes with the highest mutation frequency in high-risk (I) and low-risk (J) groups. (K) Violin plots depicted differences in immunophenoscores (IPS) between high-risk and low-risk groups. Asterisks indicate statistical significance: ***P < 0.001.

### Correlation of the risk score with TMB and immune landscape of GC

ESTIMATE algorithm was employed to explore the differences of the TME scores (stromal score, immune score and ESTIMATE score) between these two risk score groups. The results revealed that high-risk patients exhibited elevated stromal and ESTIMATE scores, indicative of an immunosuppressive phenotype, whereas low-risk patients showed immune-active features (Fig. 5A). Immune cell components analysis suggested the risk score was remarkably correlated with CAFs, hematopoietic stem cells, endothelial cells, macrophages, B cells, monocytes, myeloid dendritic cells, CD4+T cells, CD8+T cells, γ*δ*T cells, Tregs *etc*. Specifically, the risk score showed a negative correlation with CD8+ T cells (indicated by XCELL and CIBERSORT algorithms) and positive correlations with M2 macrophages (CIBERSORT-ABS and QUANTISEQ algorithms) and Treg cells (QUANTISEQ and CIBERSORT algorithms) (Fig. 5B), further supporting immune evasion in high-risk subgroups. Among SPCGs, APOD, SERPINE1, and GRP were specifically associated with immune cell subsets (Fig. S2). Notably, among them, CAFs emerged as the only cell type exhibiting significant correlations with all the three genes (Fig. 5C). Additionally, the risk score and key SPCGs (*e.g.*, SERPINE1, C6, APOD) positively correlated with the majority of immune checkpoint genes (Fig. 5D), suggesting their role in immune tolerance regulation. TMB, a potential biomarker for immunotherapy response, showed an inverse correlation with risk scores (Figs. 5E, 5F). Survival analysis confirmed TMB’s prognostic value, with high-TMB patients demonstrating superior outcomes (Fig. 5G). Integration of TMB with risk scores enhanced prognostic stratification, identifying the high-TMB/low-risk subgroup with optimal survival and low-TMB/high-risk patients with the poorest prognosis (Fig. 5H). High-risk patients exhibited significantly reduced mutation frequencies in top 20 driver genes compared to low-risk counterparts (Figs. 5I, 5J). Moreover, it has been verified that IPS value, ranged from 0 to 10, was positively bound up with tumor immunogenicity and considered to be a new predictor of immunotherapy response to anti-CTLA-4 and anti-PD-1. Our results of IPS analysis revealed that the patients in the low-risk group had higher IPS values, demonstrating a better benefit potential in immunotherapy (Fig. 5K). In general, the SPCG-based risk score system deciphers TME heterogeneity in GC, synergizes with TMB for prognostic stratification, and provides a framework for personalized immunotherapy strategies.

### Functional enrichment analysis

**Figure 6:**
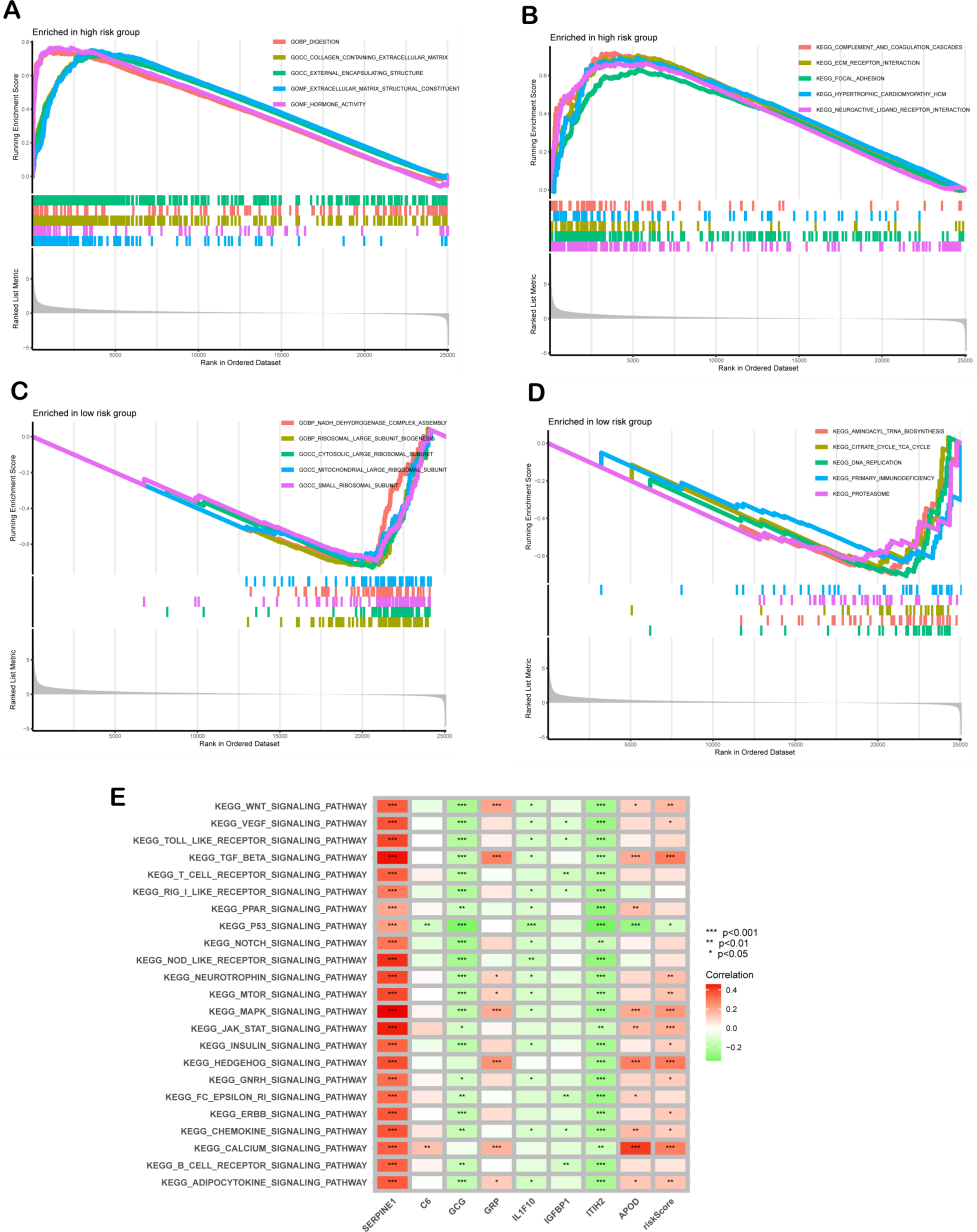
Functional enrichment analysis. (A, B) Gene Ontology (GO) enrichment (A) and The Kyoto Encyclopedia of Genes and Genomes (KEGG) pathway analyses (B) for high-risk group. (C, D) GO (C) and KEGG (D) analyses for low -risk group. (E) Correlation between risk score, individual gene in SPCGs, and KEGG signaling pathway.

GSEA revealed distinct molecular pathway activation patterns between high- and low-risk GC patients. In the high-risk group, significant enrichment was observed in extracellular matrix (ECM)-related biological processes and pro-fibrotic signaling pathways. Specifically, GO analysis highlighted activation of digestion-related processes (GOBP_DIGESTION), collagen-containing ECM organization (GOCC_COLLAGEN_CONTAINING_EXTRACELLULAR_MATRIX), and hormone activity (GOME_HORMONE_ACTIVITY) (Fig. 6A). KEGG pathway analysis further corroborated these findings, showing pronounced activation of ECM-receptor interaction, complement and coagulation cascades, and hypertrophic cardiomyopathy pathways, suggesting stromal remodeling and pathological tissue fibrosis as key features of high-risk tumors (Fig. 6B). In contrast, the low-risk group exhibited enrichment in cellular metabolism and translational regulation. GO analysis demonstrated strong involvement in mitochondrial energy production, including NADH dehydrogenase complex assembly (GOBP_NADH_DEHYDROGENASE_COMPLEX_ASSEMBLY) and ribosomal biogenesis (GOBP_RIBOSOMAL_LARGE_SUBUNIT_BIOGENESIS), with cytosolic and organellar ribosomal subunits prominently represented (Fig. 6C). KEGG analysis revealed activation of aminoacyl-tRNA biosynthesis, the citrate cycle (TCA cycle), and DNA replication pathways, indicative of heightened metabolic and proliferative homeostasis (Fig. 6D). Mechanistically, GSVA uncovered the associations between risk scores and oncogenic signaling cascades. Pro-tumorigenic pathways including TGF-β, MAPK, and Hedgehog exhibited progressive activation with increasing risk scores (Fig. 6E). Notably, SERPINE1 within the SPCG signature emerged as a multi-pathway regulatory hub, while ITIH2 exhibited inhibitory effects. These findings collectively underscore that high-risk GC is characterized by ECM-driven stromal activation, whereas low-risk tumors maintain metabolic and translational fidelity, providing mechanistic insights into their differential clinical outcomes.

**Figure 7:**
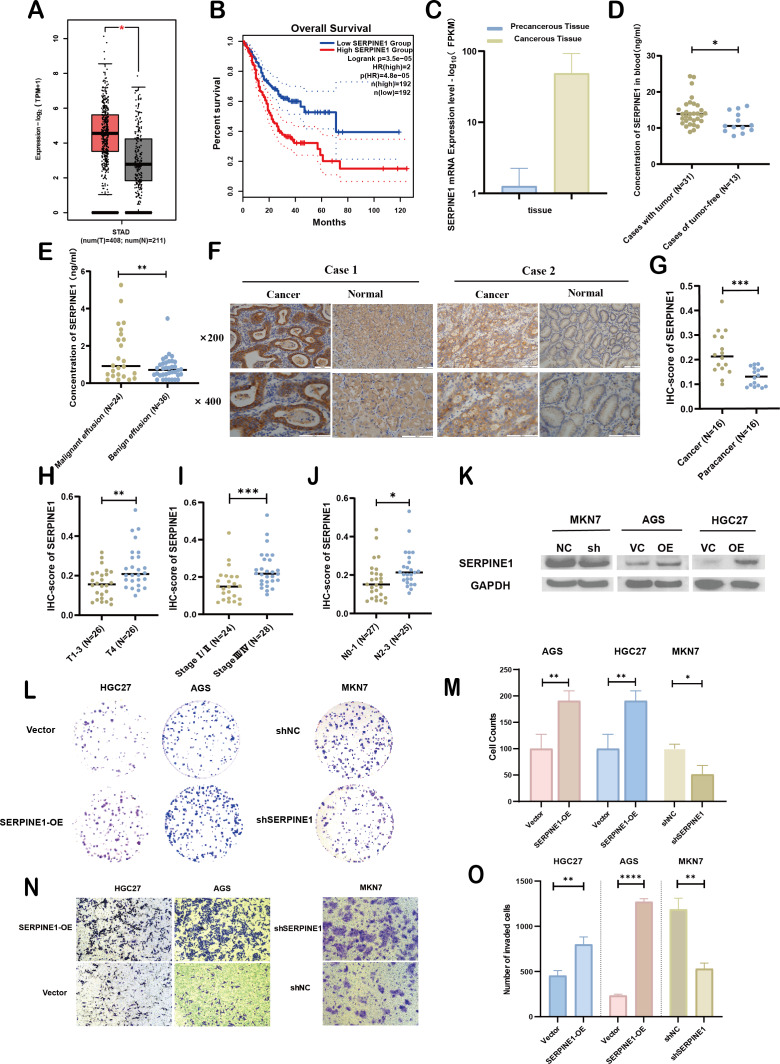
Clinical significance and bio-function of SERPINE1. (A, B) Analysis of SERPINE1 expression levels and survival outcomes using GEPIA. (C) Comparative mRNA sequencing analysis of SERPINE1 in gastric cancer *versus* adjacent non-cancerous tissues. (D, E) Quantification of SERPINE1 in peripheral blood and effusion samples *via* ELISA assay. (F, G) Immunohistochemical (IHC) analysis of SERPINE1 in matched gastric cancer and adjacent non-cancerous tissues. (H–J) Examination of the correlation between SERPINE1 expression and pathological stage, T-stage (T1-3 *vs.* T4), and N-stage. (K) Assessment of SERPINE1 protein expression levels in gastric cancer cell lines with stable downregulation and upregulation using Western Blot analysis. (L–O) Evaluation of cell proliferation and migration through colony formation and Transwell assays in cell lines with SERPINE1 overexpression and knockdown. Asterisks indicate statistical significance: *P < 0.05, **P < 0.01, ***P < 0.001, ****P < 0.0001.

### Experimental validation of SERPINE1’s clinical significance and biological function in GC

To assess the expression levels and clinical relevance of SERPINE1 in GC, we utilized the Gene Expression Profiling Interactive Analysis (GEPIA) platform (http://gepia.cancer-pku.cn/index.html). Our analysis revealed significantly higher expression of SERPINE1 in GC tissues compared to normal tissues (Fig. 7A). Kaplan–Meier survival analysis further demonstrated that elevated SERPINE1 expression was associated with poorer prognosis in GC patients (Fig. 7B). To further validate the clinical relevance of SERPINE1, we analyzed clinical specimens from our institutional cohort. Next-generation sequencing of paired post-surgical tissues demonstrated substantially higher SERPINE1 mRNA levels in tumor tissues compared to adjacent precancerous lesions (Fig. 7C; Table S1). Circulating SERPINE1 protein levels, as determined by ELISA, showed progressive elevation in advanced GC patients compared to post-operative tumor-free controls (Fig. 7D; clinical details in Table S2). Notably, malignant effusions exhibited significantly higher SERPINE1 concentrations than benign effusions (Fig. 7E; Table S3). Immunohistochemical analysis of 52 GC specimens (including 16 matched tumor-adjacent pairs) revealed pronounced SERPINE1 overexpression in malignant tissues (Figs. 7F, 7G; Table S4). Subgroup analysis demonstrated significantly elevated SERPINE1 immunoreactivity scores in advanced pathological stages (III *vs.* I–II), deeper tumor invasion (T4 *vs.* T1-3), and extensive nodal metastasis (N2-3 *vs.* N0-1) (Figs. 7H–7J). However, no significant associations were observed with tumor differentiation status or histological subtypes (Fig. S3). The robustness of these findings was confirmed by sensitivity analyses, which showed that all key associations remained statistically significant after excluding outliers (Fig. S4).

We assessed the basal expression of SERPINE1 by RT-qPCR and measured its secretory levels in the cell culture supernatant by western blot and ELISA in the gastric cancer cell lines and normal gastric epithelial cells used in this study. The results confirmed that cell lines with high SERPINE1 expression secreted higher levels of the protein, while those with low expression exhibited lower secretory levels (Fig. S5). To elucidate SERPINE1’s functional role, we established genetic manipulation models based on its expression patterns across cell lines. Given the lower baseline expression of SERPINE1 in AGS and HGC27 cells and its higher expression in MKN7 cells, we generated SERPINE1-knockdown MKN7 cells (shSERPINE1) and SERPINE1-overexpressing AGS/HGC27 lines (SERPINE1-OE). Successful modulation was confirmed at both transcriptional and protein levels (Fig. 7K; Fig. S6). Functional assays demonstrated that SERPINE1 overexpression significantly enhanced colony formation capacity and transwell invasion, while SERPINE1 knockdown produced opposing effects (Figs. 7L–7O).

Overall, these findings suggest that SERPINE1 may promote GC cell proliferation and invasion *in vitro*. Elevated SERPINE1 levels appear to be associated with advanced clinicopathological stages and poorer prognosis in GC patients, supporting its further investigation as a potential therapeutic target and biomarker for this disease.

### Interplay between CAFs and SERPINE1 in GC progression

**Figure 8:**
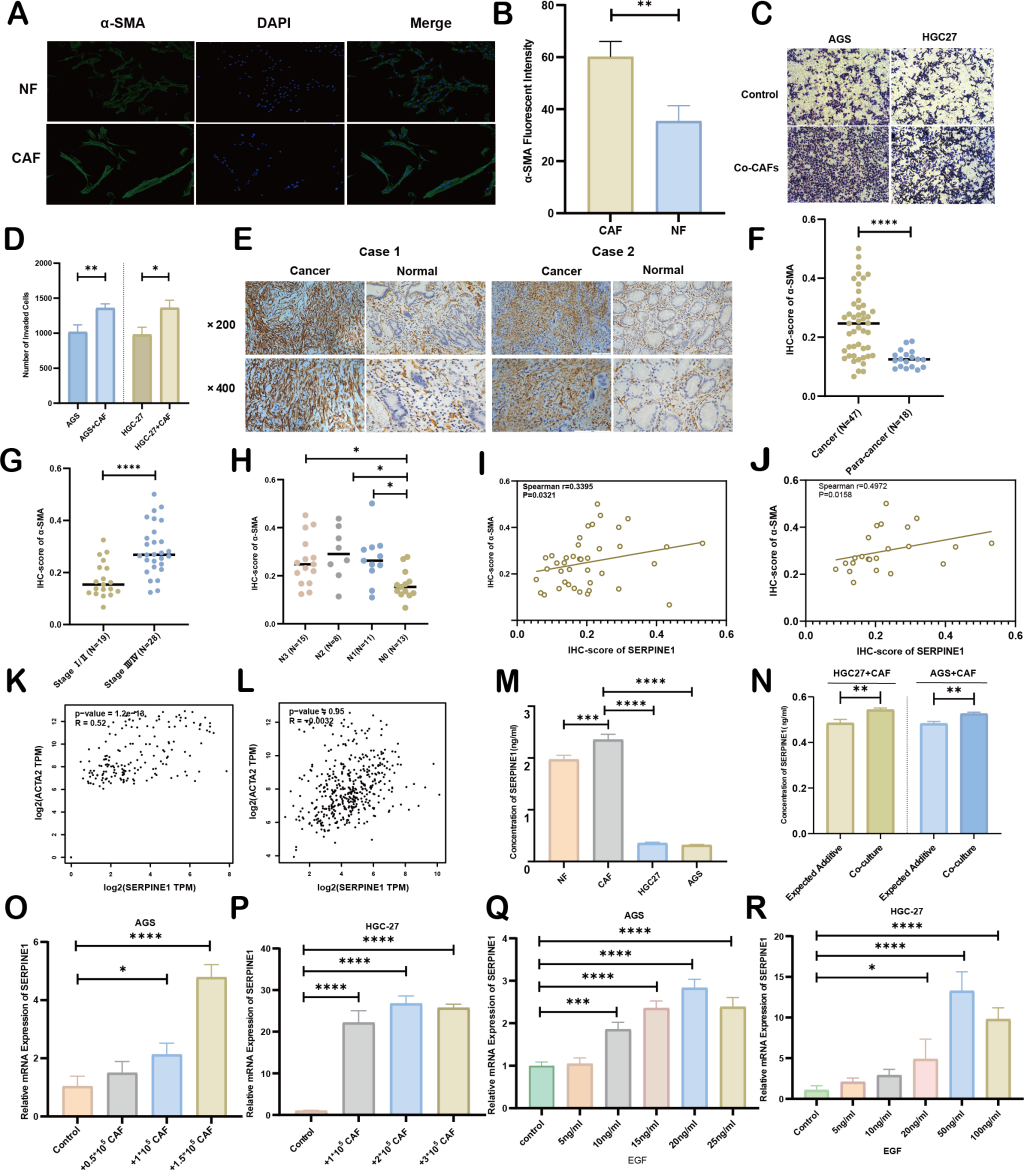
Association between CAFs and SERPINE1. (A, B) α-SMA was more expressed in CAFs than NFs, indicated by immunofluorescence. (C, D) Co-culture with CAFs enhance d GC cell invasion, demonstrated by a Transwell assay. (E, F) IHC staining revealed higher α- SMA expression in cancer tissues compared to para-cancer tissues. (G, H) IHC-score analysis demonstrated a correlation between α- SMA expression levels and TNM staging. (I, J) IHC analysis showed a positive correlation between the expression of SERPINE1 and α- SMA. (K, L) Correlation analysis using the GEPIA platform confirmed a positive association between SERPINE1 and ACTA2 expression in both TCGA databases (K) and GTEx database (L). (M) ELISA results suggesting CAFs secreted much more SERPINE1 than NFs and GC cell lines. (N) Co-culture of gastric cancer cells and CAFs synergistically enhances SERPINE1 secretion. (O, P) mRNA level of SERPINE1 significantly increased in GC cells upon co-culture with CAFs by RT-q PCR. (Q, R) The addition of epidermal growth factor (EGF) led to an upregulation of SERPINE1 expression in GC cells. CAFs, cancer-associated fibroblasts. NF, normal fibroblasts. EGF, epidermal growth factor. Asterisks indicate statistical significance: *P < 0.05, **P < 0.01, ***P < 0.001, ****P < 0.0001.

As principal components of tumor stroma, CAFs were identified using α-SMA—a canonical marker for CAFs, which is encoded by the ACTA2 gene. Immunofluorescence analysis confirmed significantly enhanced α-SMA expression in CAFs compared to NFs derived from our primary cultures (Figs. 8A, 8B). Functional characterization through Transwell assays demonstrated that CAFs substantially potentiated the invasive capacity of HGC27 and AGS cells (Figs. 8C, 8D). Clinical validation using 47 GC specimens revealed pronounced α-SMA elevation in tumor tissues *versus* matched adjacent normal tissues (Figs. 8E, 8F; Table S5). Subgroup analyses identified significant associations between α-SMA overexpression and advanced disease stage (III *vs.* I–II) as well as lymph node metastasis (N1-3 *vs.* N0) (Figs. 8G, 8H). However, no correlations were observed with T-stage invasion depth or histopathological subtypes (Fig. S7). Correlative analysis of 40 GC specimens demonstrated significant positive correlation between SERPINE1 and α-SMA expression (*r* = 0.3395, *p* = 0.0321; Fig. 8I), which intensified in stage III patients (*r* = 0.4972, *p* = 0.0158; Fig. 8J). Exclusion of outliers further strengthened the association between SERPINE1 and α-SMA IHC scores, yielding higher correlation coefficients (*r* = 0.4054 for all patients; *r* = 0.4972 for stage III) and greater statistical significance (*p* = 0.0142 and *p* = 0.0158, respectively; Fig. S4). These findings were corroborated by GEPIA analysis showing SERPINE1-ACTA2 co-expression in both TCGA (Fig. 8K) and GTEx cohorts (Fig. 8L). ELISA quantification revealed that CAFs secreted higher SERPINE1 compared to NFs and GC cell lines (Fig. 8M). Furthermore, when HGC27 or AGS cells were co-cultured with CAFs, the SERPINE1 concentration in the supernatant significantly exceeded the theoretical additive concentration derived from the corresponding monocultures (Fig. 8N), indicating a synergistic enhancement. Notably, co-culture experiments uncovered a dose-dependent upregulation of SERPINE1 in HGC27 and AGS cells upon CAFs exposure (Figs. 8O, 8P). Intriguingly, exogenous epidermal growth factor (EGF) stimulation—a canonical ERBB pathway activator–mimicked this effect, significantly elevating SERPINE1 expression in both cell lines (Figs. 8Q, 8R). This observation aligns with prior KEGG pathway analysis linking SERPINE1 to ERBB signaling (Fig. 6E). These findings suggest that CAFs may serve as potential contributors to SERPINE1-driven malignancy, possibly through direct secretion and EGF/ERBB-mediated paracrine mechanisms. This stromal-epithelial interaction represents an avenue worthy of further exploration for future therapeutic strategies.

## Discussion

GC is characterized by profound intratumoral heterogeneity, and current molecular markers exhibit limited utility in predicting prognosis and guiding stratified treatment in clinical practice (Joshi & Badgwell, 2021). While next-generation sequencing has enabled proliferation of tissue-based gene signatures for GC outcome prediction (Cheong et al., 2022; Mak et al., 2022; Zeng et al., 2019; Zhai et al., 2023), their clinical translation remains constrained by tissue acquisition invasiveness and analytical complexity. Liquid biopsy approaches based on circulating tumor DNA (ctDNA) also face limitations, including low ctDNA abundance in plasma and a lack of GC-specific validation (Zhang et al., 2022b; Ma et al., 2023). In this context, we developed a non-invasive SPCG signature that combines prognostic assessment with TME characterization. The 8-SPCG model represents an exploratory approach with several potential strengths: (1) utilization of systemically detectable secreted proteins rather than tissue-dependent markers; (2) simultaneous evaluation of clinical outcomes and immune landscape features; and (3) functional emphasis on SERPINE1 as a potential mediator connecting CAFs to malignant progression. These aspects may provide a basis for further development of secreted protein-based biomarkers in GC.

Secreted proteins—such as cytokines, growth factors, and hormones—are involved in key tumor-related processes including inflammation, proliferation, metastasis, and immune regulation, rendering them candidate biomarkers and therapeutic targets (Pavlou & Diamandis, 2010; Brown et al., 2013). Despite their potential, the functional roles of the secreted proteome in GC remain incompletely characterized (Repetto et al., 2023; Marimuthu et al., 2013). Although previous proteomic studies have identified tissue- or blood-based biomarkers for diagnosis and prognosis (Gu et al., 2023; Li et al., 2022a; Abramowicz et al., 2015; Feng et al., 2024; Min et al., 2020; Song et al., 2020; Vizeacoumar et al., 2021; Zheng et al., 2022), few have incorporated TME interactions or therapy response prediction (Huang et al., 2021; Li et al., 2022c). To address this gap, we constructed an 8-SPCG prognostic signature using integrative bioinformatics and experimental validation. The signature showed suggestive prognostic value (5-year AUC: 0.745) and was associated with TME features, particularly CAFs infiltration. A nomogram incorporating clinicopathological variables further supported risk stratification.

Methodologically, our transcriptome-based approach for studying secreted proteins offers a potential alternative that may overcome several limitations inherent to conventional proteomic techniques—such as ELISA or mass spectrometry (Li et al., 2022a; Huang et al., 2021; Subbannayya et al., 2015),—which are often constrained by antibody availability, high cost, and technical complexity (Repetto et al., 2023; Birhanu, 2023; Krieg et al., 2020). By analyzing RNA-seq data from TCGA-STAD to identify circulating protein-encoding genes, we developed a tractable mRNA-based signature.This approach has been less commonly employed in GC biomarker discovery.

The 8-SPCG signature integrates molecules with diverse functional roles: SERPINE1, a metastasis-promoter linked to poor prognosis (Lv et al., 2023; Vachher et al., 2020; Liao et al., 2018; Xu et al., 2022; Chen et al., 2022b); C6, a complement cascade component mediating membrane attack complex formation; GRP, a cytokine inducer activating fibro-inflammatory pathways (Sun et al., 2023); GCG, a metabolic regulator enhancing chemotherapy sensitivity (Xu et al., 2024); IL1F10 (IL38), an inflammation modulator with antitumor potential (Silva et al., 2024); IL1F10 (IL38), an inflammation modulator with antitumor potential (Silva et al., 2024); IGFBP1, an oncogenic behavior regulator (Lin et al., 2021); ITIH2, an extracellular matrix stabilizer (Zhang et al., 2024); and APOD, a multifunctional protein influencing lipid metabolism and oxidative stress (Rassart et al., 2000). This functional diversity suggests the signature may capture aspects of complex tumor-stroma interactions.

Our multi-dimensional analysis supported SERPINE1’s oncogenic role in gastric carcinogenesis. IHC, mRNA sequencing, and ELISA indicated higher SERPINE1 expression in GC tissues compared with paracancerous samples, in peripheral blood relative to post-operative samples, and in malignant effusions *versus* benign controls, with elevated levels correlating with advanced TNM stage. Further functional assays suggested that SERPINE1 promotes proliferation, migration, and invasion in GC cells. Previous studies have linked SERPINE1 to biological processes, such as DNA repair and regulation (Wei et al., 2023; Su et al., 2023), hypoxia response (Zhang et al., 2023), angiogenesis (Hong et al., 2022; Zhang, Lei & Jing, 2020), endothelial-to-mesenchymal transition (EMT) (Jiang et al., 2020; Hu et al., 2021), and multiple signaling pathways including MEK/ERK, NF-κB, TGF, *etc* (Wang et al., 2023). Our KEGG and GO analyses also linked SERPINE1 to key pathways such as Wnt, VEGF, TGF, JAK-STAT, mTOR, MAPK, and ERBB. *In vitro*, SERPINE1 expression appeared EGF-dependent in GC cells, consistent with reports in keratinocytes and other solid tumors (Freytag et al., 2010; Alberti et al., 2012; Song, Thalacker & Nilsen-Hamilton, 2012; Wilkinsport et al., 2009). Nevertheless, the function of SERPINE1 within the GC TME deserves further study.

The 8-SPCG risk model showed the potential to stratify TME characteristics in our analysis. Patients with high-risk scores exhibited an immunosuppressive TME phenotype, characterized by CAFs enrichment, reduced CD8+ T cells, elevated Tregs and M2 macrophages, higher expression of immune checkpoint genes, and decreased TMB and IPS—all indicative of a poorer likelihood of response to immunotherapy. Functional analysis showed the high-risk group was enriched in ECM processes and pro-fibrotic pathways, aligning with CAF-driven stromal remodeling observed in single-cell studies (Li et al., 2022b; Luo et al., 2022). CAFs are known to interact with immune and tumor cells to support tumor growth, epithelial-mesenchymal transition, and therapy resistance (Li et al., 2022b; Luo et al., 2022; Zhang et al., 2020). In line with this, we found that α-SMA, a CAFs marker, was overexpressed in GC tissues and associated with unfavorable outcomes. Furthermore, GC cells co-cultured with CAFs acquired enhanced migratory capability, supporting the functional role of CAFs in promoting tumor aggression. Although CAFs are understood to secrete bioactive factors that promote tumor progression (Zhang et al., 2022a; Lu et al., 2023), their interplay with SERPINE1 in GC remains poorly defined. Our data suggest possible CAF–SERPINE1 crosstalk, evidenced by: (1) CAFs exhibited higher SERPINE1 secretion than normal fibroblasts; (2) co-culture with CAFs induced SERPINE1 upregulation in GC cells; and (3) a positive correlation was observed between α-SMA and SERPINE1 expression in GC patients, especially in those with stage III disease. These findings position SERPINE1 as a potential stromal-epithelial mediator, though the detailed signaling mechanisms (particularly EGF-ERBB axis involvement) require further exploration.

Several limitations warrant consideration. First, the generalizability of the findings may be constrained by the modest cohort size and considerable heterogeneity in molecular expression levels. Second, while SERPINE1 was functionally characterized, the roles of other signature components (*e.g.*, C6, GRP, GCG) in gastric cancer remain unclear and merit further investigation. Third, the retrospective design of our clinical analysis necessitates prospective validation in larger, independent cohorts to verify the prognostic utility of the model and the biological role of SERPINE1. Finally, the precise molecular mechanisms underlying CAF-SERPINE1 interactions, particularly their impact on therapeutic resistance, remain to be fully elucidated.

## Conclusions

In this study, we developed a non-invasive prognostic signature based on SPCGs for GC. The signature showed potential in stratifying patients into risk subgroups with differing survival outcomes and tumor microenvironment features. Our functional data underscore the likely importance of SERPINE1 in GC progression and its interplay with cancer-associated fibroblasts (CAFs), supporting its further investigation as a candidate biomarker and therapeutic target. These findings contribute to a growing understanding of how tumor-secreted factors shape the TME, and may provide a basis for future research toward clinical translation.

## Supplemental Information

10.7717/peerj.20517/supp-1 Supplemental Information 1 Original western blot mages for Fig. 7J (A–F) and Supplementary Fig. 5B (G)

10.7717/peerj.20517/supp-2 Supplemental Information 2 The correlation of APOD, SERPINE 1 and GRP with immune cells in GC

10.7717/peerj.20517/supp-3 Supplemental Information 3 The correlation of SERPINE1 with pathological characteristics of GC

10.7717/peerj.20517/supp-4 Supplemental Information 4 Sensitivity analyses

10.7717/peerj.20517/supp-5 Supplemental Information 5 Analysis of SERPINE1 expression in cell lines

10.7717/peerj.20517/supp-6 Supplemental Information 6 Validation of stable cell lines by RT-PCR

10.7717/peerj.20517/supp-7 Supplemental Information 7 The correlation of α- SMA with pathological characteristics of GC

10.7717/peerj.20517/supp-8 Supplemental Information 8 Original microscopy images for Figures 7G and 7N

10.7717/peerj.20517/supp-9 Supplemental Information 9 Original microscopy images for Figures 8A, 8C and 8E

10.7717/peerj.20517/supp-10 Supplemental Information 10 Differential expression of SERPINE1 in gastric cancer and matched adjacent tissues via RNA-Seq: raw data

10.7717/peerj.20517/supp-11 Supplemental Information 11 Clinical and pathological data of GC patients of advanced stage with tumor and postsurgical cases of tumor-free state

10.7717/peerj.20517/supp-12 Supplemental Information 12 Clinical data of patients with malignant and benign effusion

10.7717/peerj.20517/supp-13 Supplemental Information 13 Clinical and pathological information of 52 GC cases available for immunohistochemical data of SERPINE1 in our center

10.7717/peerj.20517/supp-14 Supplemental Information 14 Clinical and pathological information of 47 GC cases available for immunohistochemical data of α-SMA in our center

10.7717/peerj.20517/supp-15 Supplemental Information 15 SERPINE1 levels in pleural and peritoneal effusions measured by ELISA (ng/ml) Comparative analysis of SERPINE1 protein in malignant vs. non-malignant effusions: raw ELISA data from pleural and peritoneal fluids.

10.7717/peerj.20517/supp-16 Supplemental Information 16 SERPINE1 levels in peripheral blood of GC patients measured by ELISA (ng/ml) Comparative serum SERPINE1 levels in gastric cancer: raw ELISA data of tumor - bearing vs. tumor-free cohorts.

10.7717/peerj.20517/supp-17 Supplemental Information 17 SERPINE1 concentration in supernatants of CAF, NF, AGS, and HGC27 Cells via ELISA

10.7717/peerj.20517/supp-18 Supplemental Information 18 mRNA level of SERPINE1 in AGS cells upon co-culture with CAFs by RT- qPCR Raw RT-qPCR data.

10.7717/peerj.20517/supp-19 Supplemental Information 19 mRNA level of SERPINE1 in HGC27 cells upon co-culture with CAFs by RT- qPCR Raw RT-qPCR data.

10.7717/peerj.20517/supp-20 Supplemental Information 20 Dose-dependent regulation of SERPINE1 mRNA by EGF in HGC27 Cells Raw RT-qPCR data.

10.7717/peerj.20517/supp-21 Supplemental Information 21 Dose-dependent regulation of SERPINE1 mRNA by EGF in AGS Cells Raw RT-qPCR data.

10.7717/peerj.20517/supp-22 Supplemental Information 22 mRNA level of SERPINE1 in AGS cells with stable SERPINE1 up regulation by RT-qPCR Raw RT-qPCR data.

10.7717/peerj.20517/supp-23 Supplemental Information 23 mRNA level of SERPINE1 in HGC27 cells with stable SERPINE1 up regulation by RT-qPCR Raw RT-qPCR data.

10.7717/peerj.20517/supp-24 Supplemental Information 24 mRNA level of SERPINE1 in MKN7 cells with stable SERPINE1 knockdown by RT-qPCR Raw RT-qPCR data.

10.7717/peerj.20517/supp-25 Supplemental Information 25 MIQE checklist

10.7717/peerj.20517/supp-26 Supplemental Information 26 R code used for the bioinformatics analysis

## Additional Information and Declarations

### Data Availability

The following information was supplied regarding data availability: The raw data are available in the Supplementary Files.
