# Supplementary figures and images for: A non-invasive secreted protein-based gene signature for prognostic stratification and tumor microenvironment assessment in gastric cancer
Source: PeerJ. 2026 Jan 13;14:e20517. doi: 10.7717/peerj.20517 (PMC12810363; doi:10.7717/peerj.20517)

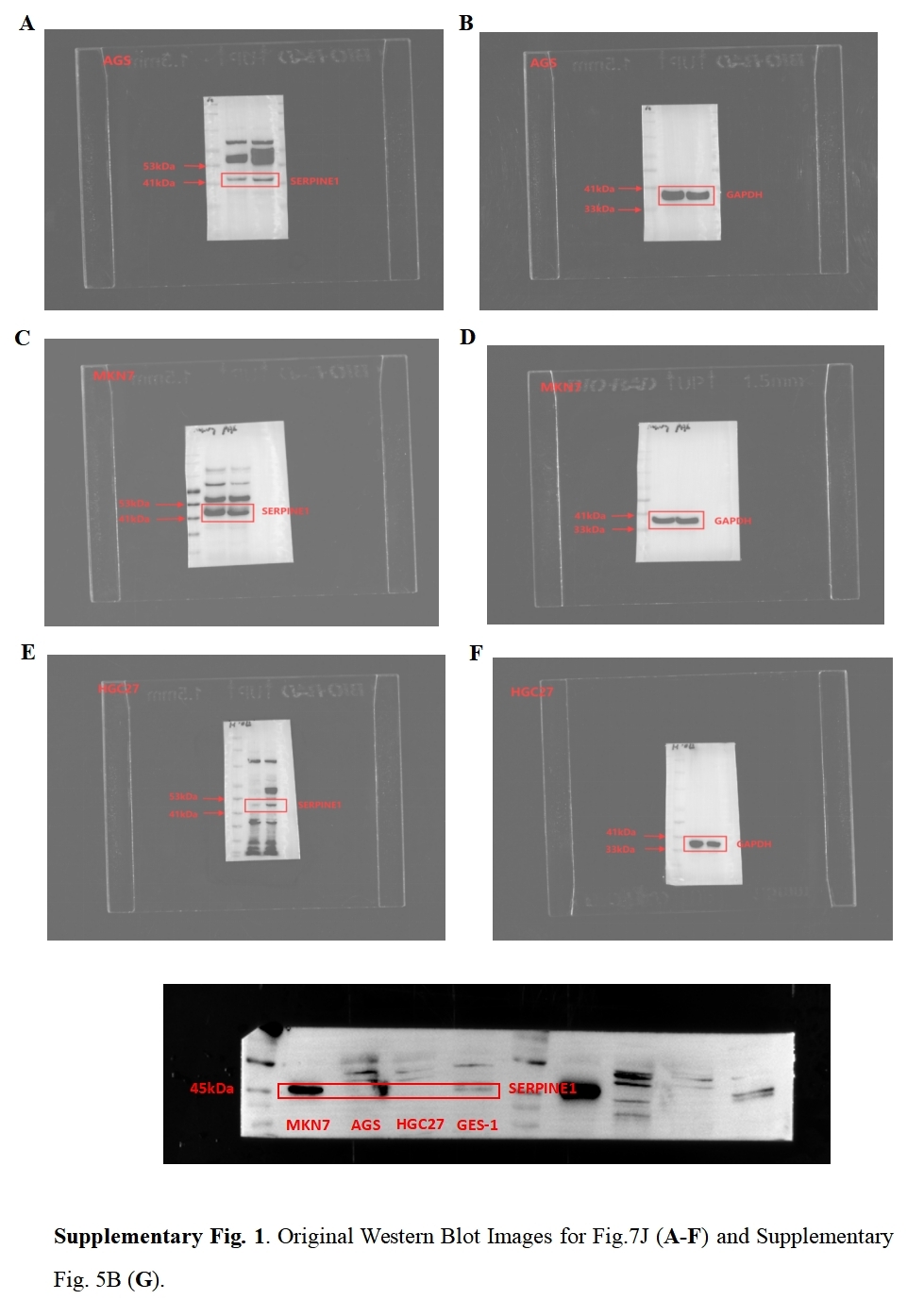

Supplement: Supplemental Information 1 [file peerj-14-20517-s001.jpg]

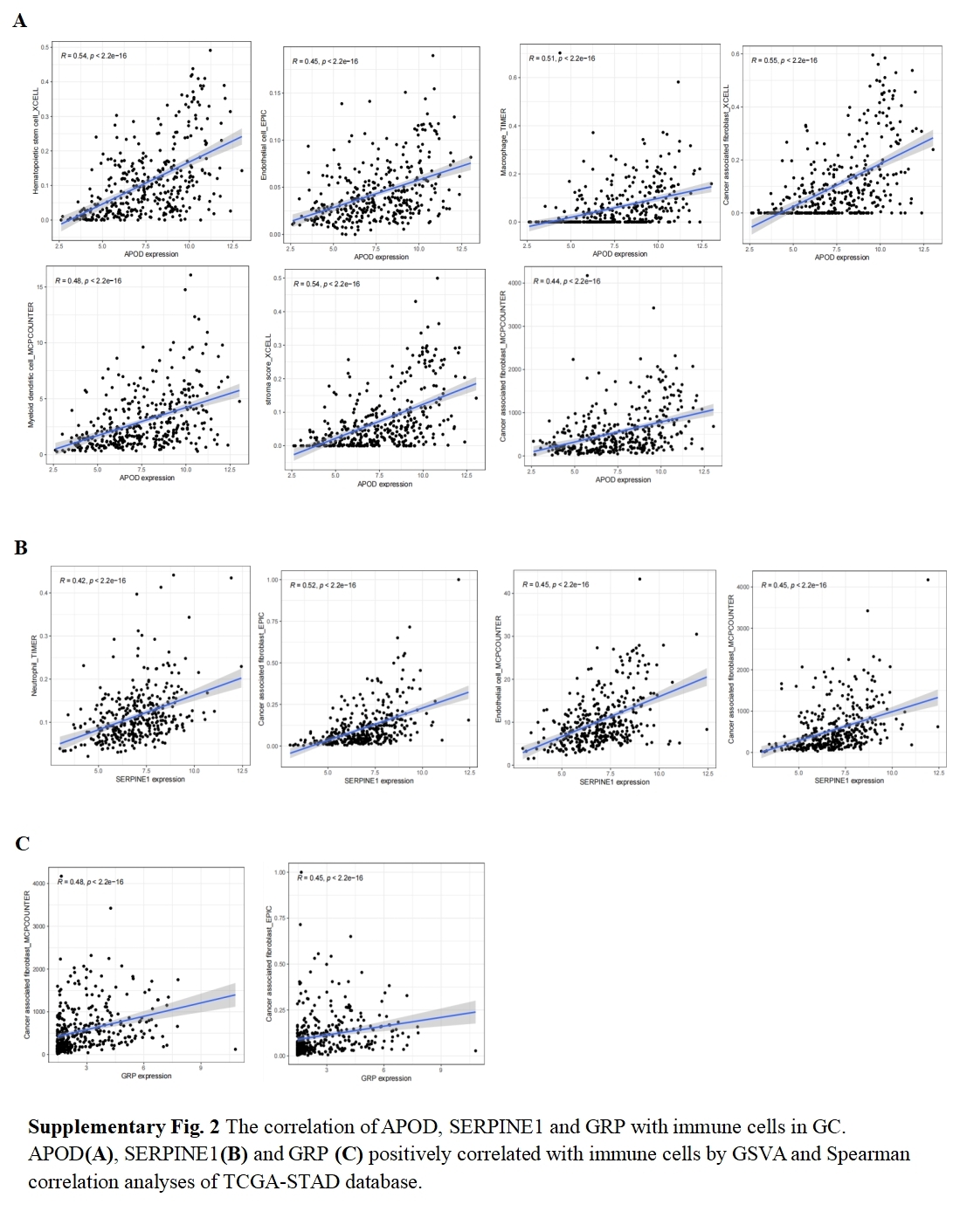

Supplement: Supplemental Information 2 [file peerj-14-20517-s002.jpg]

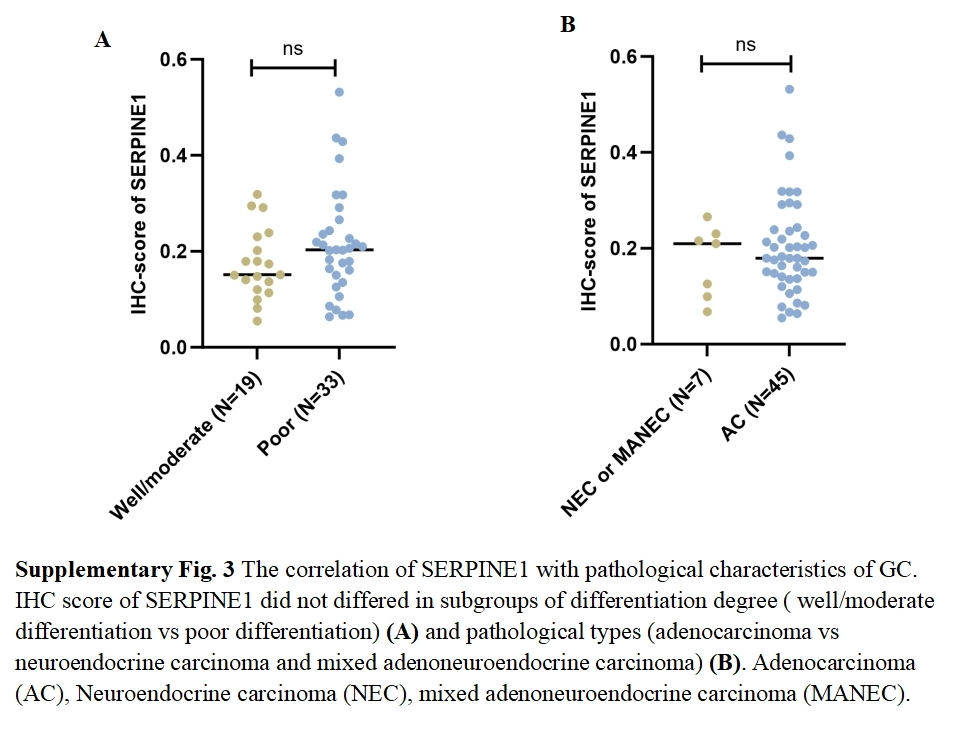

Supplement: Supplemental Information 3 [file peerj-14-20517-s003.jpg]

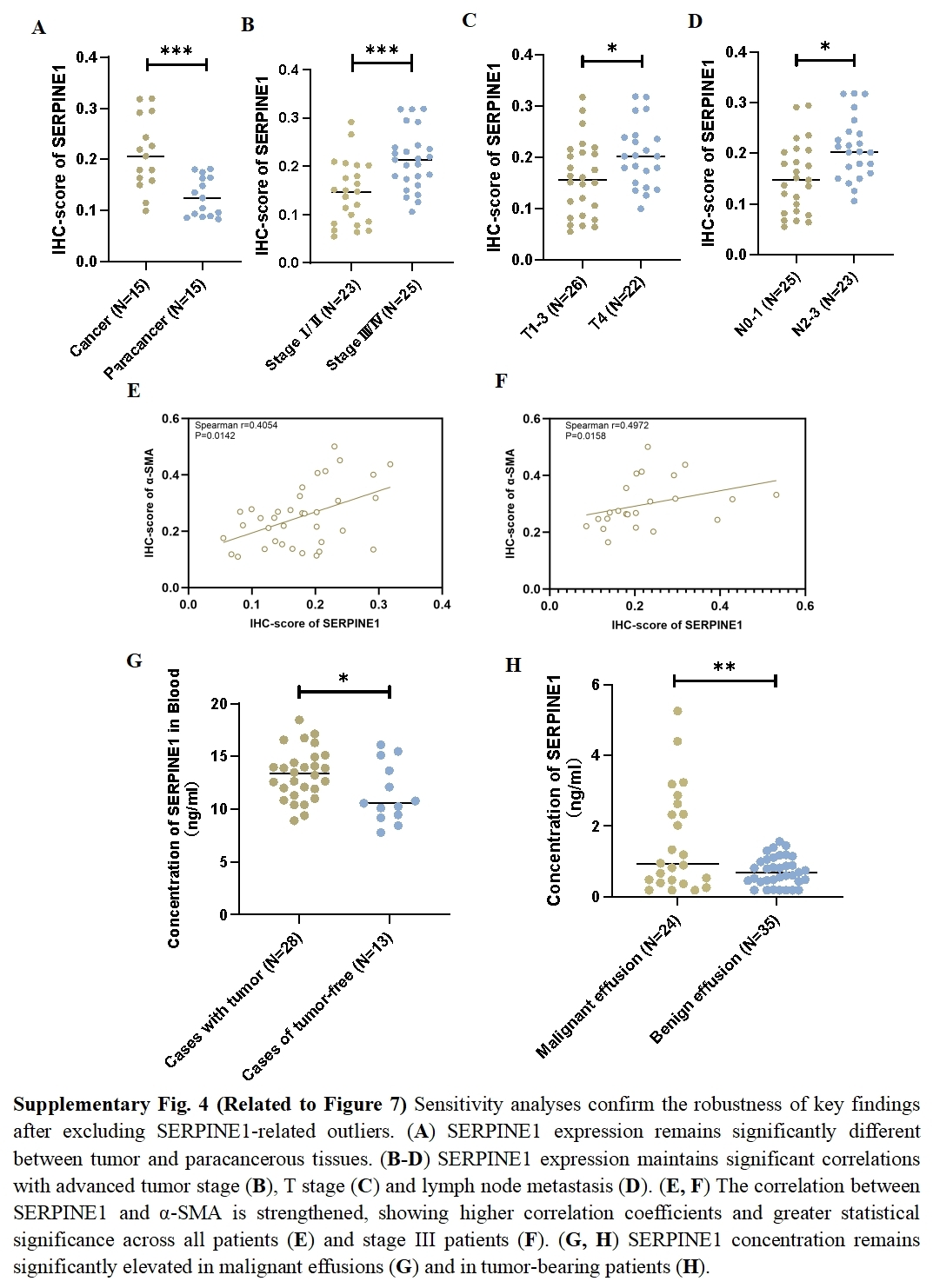

Supplement: Supplemental Information 4 [file peerj-14-20517-s004.jpg]

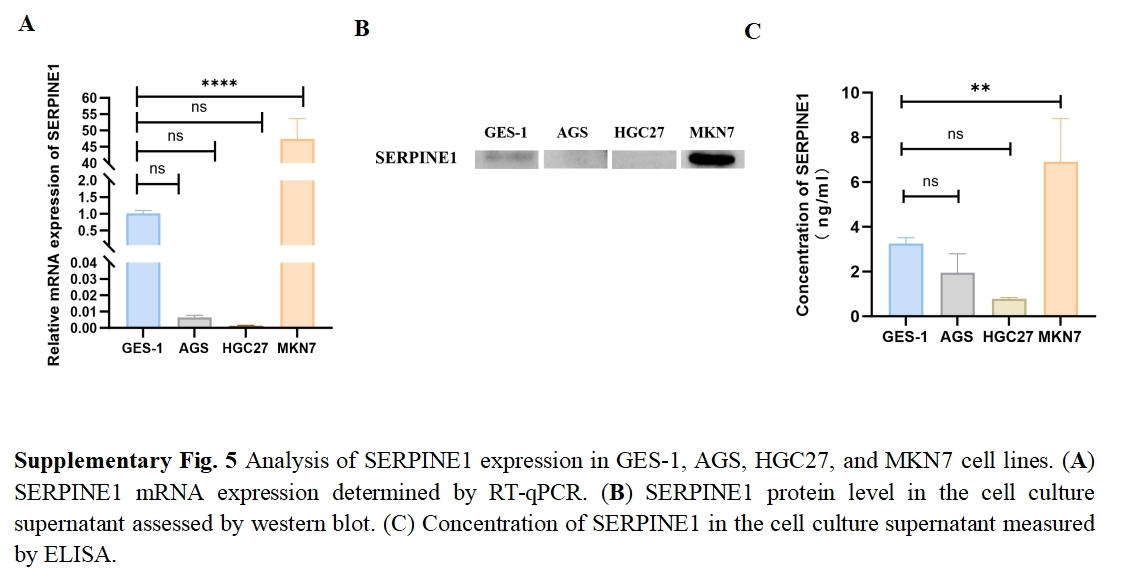

Supplement: Supplemental Information 5 [file peerj-14-20517-s005.jpg]

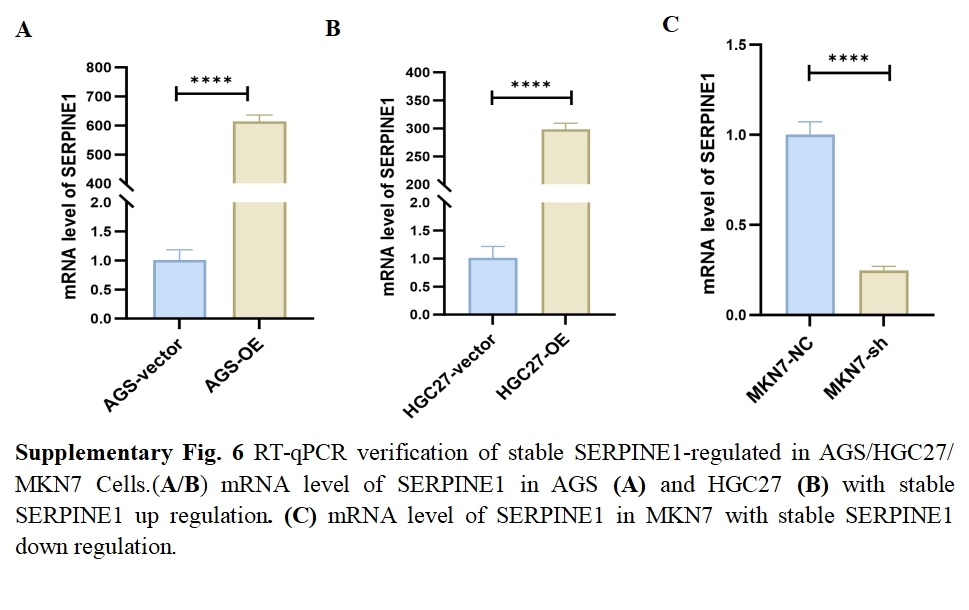

Supplement: Supplemental Information 6 [file peerj-14-20517-s006.jpg]

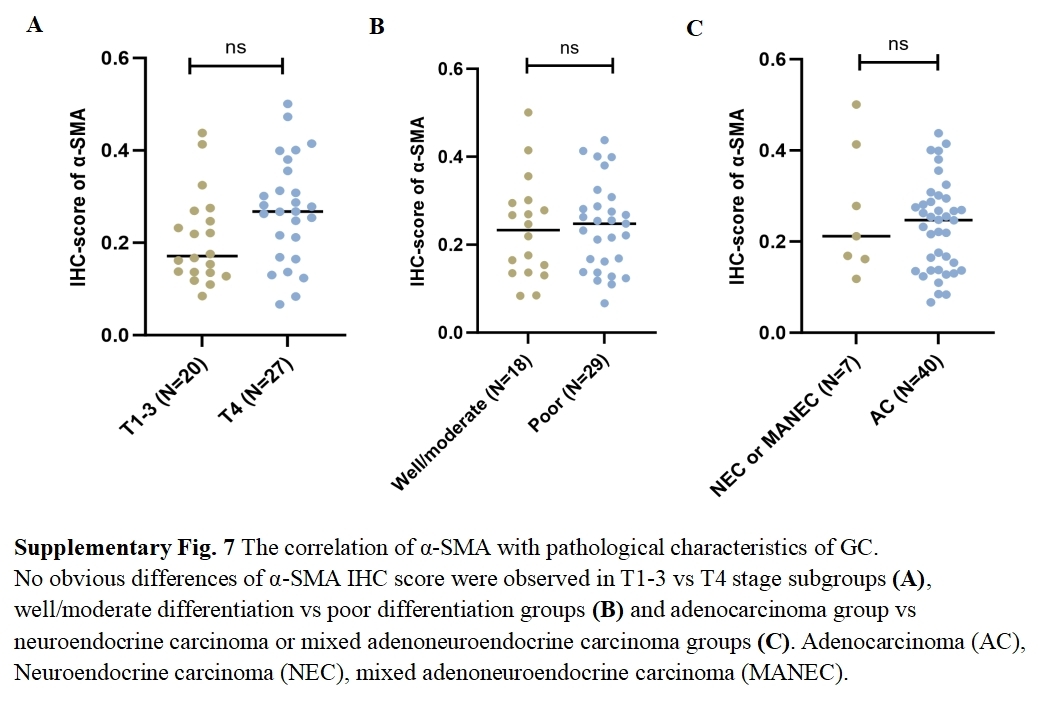

Supplement: Supplemental Information 7 [file peerj-14-20517-s007.jpg]

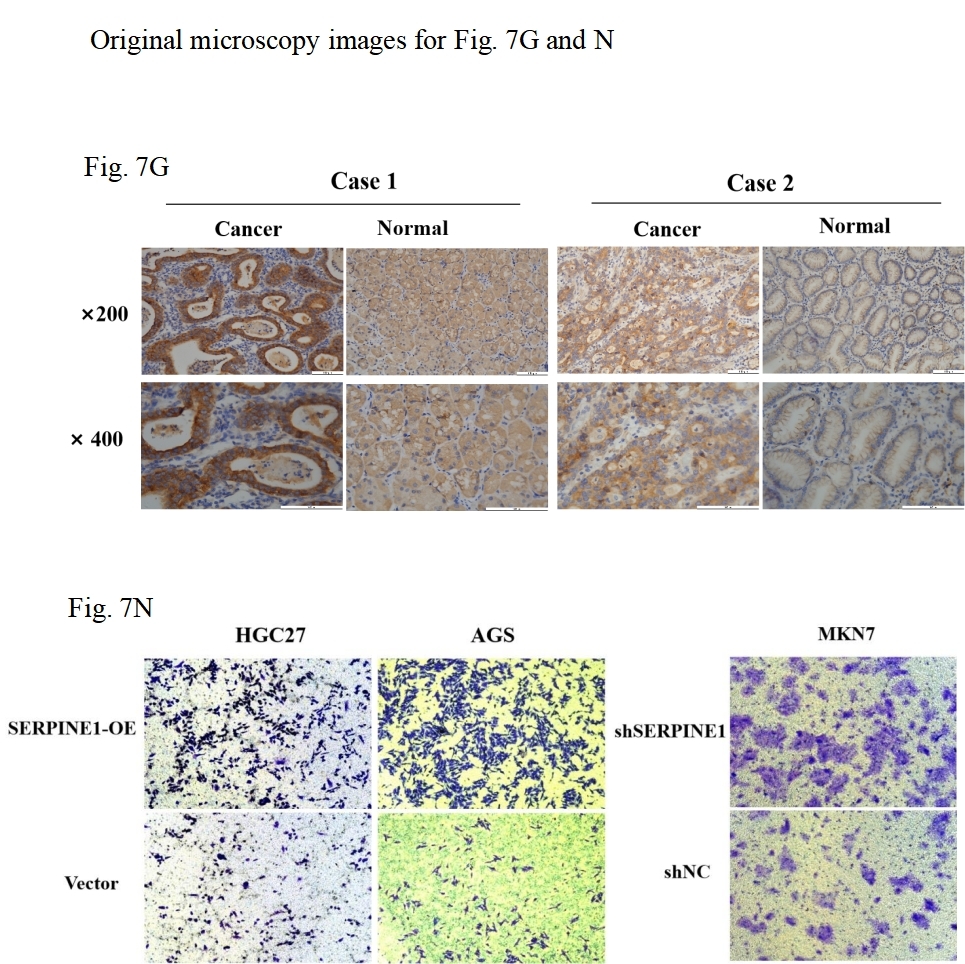

Supplement: Supplemental Information 8 [file peerj-14-20517-s008.jpg]

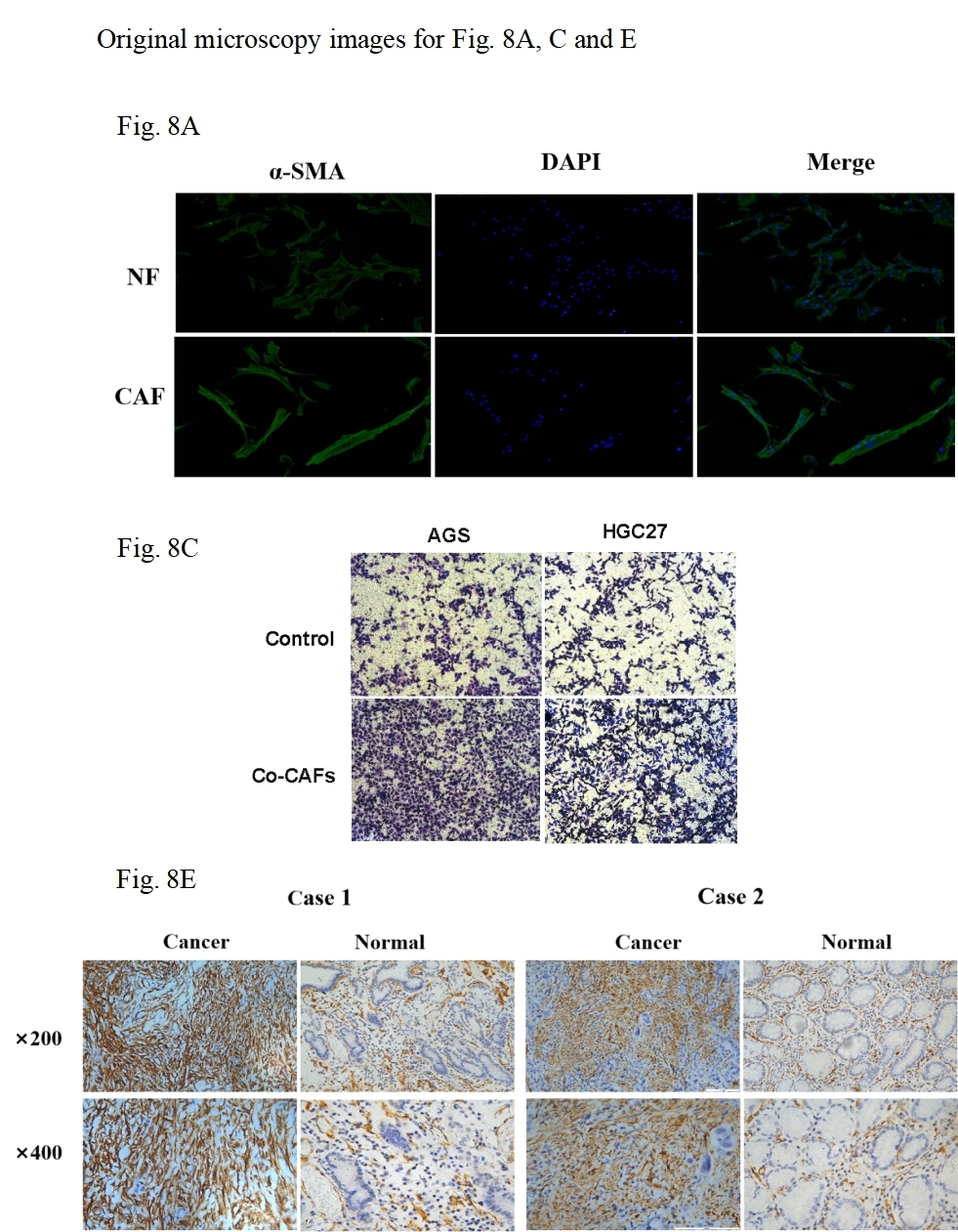

Supplement: Supplemental Information 9 [file peerj-14-20517-s009.jpg]
