# Supplementary material for: A non-invasive secreted protein-based gene signature for prognostic stratification and tumor microenvironment assessment in gastric cancer
Source: PeerJ. 2026 Jan 13;14:e20517. doi: 10.7717/peerj.20517 (PMC12810363; doi:10.7717/peerj.20517)
Supplement: Supplemental Information 11 [file peerj-14-20517-s011.docx]

| **Supplementary Table 2** Clinical and pathological data of GC patients of advanced stage with tumor and postsurgical cases of tumor-free state. | | | | |
| --- | --- | --- | --- | --- |
| Characteristics | | Cases with tumor  ( N=31) | Cases of tumor-free (N=13) | *P value* |
| Age（medium ± SD） | | 68.1±11.99 | 66.46±7.785 | 0.6538 |
| Gender | Male | 26 (83.87%) | 13 (100%) | 0.3005 |
|  | Female | 5 (16.13%) | 0 |  |
| Pathological types | Adenocarcinoma | 31 (100%) | 11 (84.61%) | 0.0825 |
|  | Adenoneuroendocrine carcimona | 0 | 2 (15.38%) |  |
| Grade | Well/moderate | 8 (25.80%) | 2 (15.38%) | 0.7355 |
|  | Poor | 17 (54.84%) | 9 (69.23%) |  |
|  | Unknown | 6 (19.35%) | 2 (15.38%) |  |
| Tumor stage | Ⅰ | 0 | 1 (7.69%) | / |
|  | Ⅱ | 0 | 3 (23.08%) |  |
|  | Ⅲ | 2 (6.45%) | 8 (61.54%) |  |
|  | Ⅳ | 29 (93.55%) | 1 (7.69%) |  |
