# Supplementary material for: A non-invasive secreted protein-based gene signature for prognostic stratification and tumor microenvironment assessment in gastric cancer
Source: PeerJ. 2026 Jan 13;14:e20517. doi: 10.7717/peerj.20517 (PMC12810363; doi:10.7717/peerj.20517)
Supplement: Supplemental Information 12 [file peerj-14-20517-s012.docx]

| **Supplementary Table 3** Clinical  data of patients with malignant and benign effusion | | | | |
| --- | --- | --- | --- | --- |
| Characteristics | | Malignant effusion (N=26) | Benign effusion (N=45) | P value |
| Excluded cases | | 1 case with cerebrospinal fluid sample; 1 case with venous thrombosis | 5 case with cerebrospinal fluid sample; 2 case with venous thrombosis;1 case with hemorrhagic event;1case diagnosed with peritoneal metastasis by laparotomy | / |
| Included cases | | N=24 | N=36 |  |
| Age（medium ± SD） | | 66.75 ± 11.33 | 68.78 ± 16.26 | 0.5978 |
| Gender | Male | 7 (29.17%) | 23 (63.89%) | 0.0169 |
|  | Female | 17 (70.83%) | 13 (36.11%) |  |
| Primary diseases | | Lung cancer  (12, 50%) | Infectious disease  (13, 36.11%,) | / |
|  |  | Gastric cancer  (5, 20.83%) | Tuberculous serositis  (7, 19.44%) |  |
|  |  | Ovarian cancer  (4, 16.67%) | Cirrhosis  (5, 13.89%) |  |
|  |  | Breast cancer  (1, 4.17%) | Hypoproteinemia  (4, 11.11%) |  |
|  |  | Pancreatic cancer  (1, 4.17%) | Heart failure  (2, 5.56%) |  |
|  |  | Malignant neoplasm of unknown primary  (1, 4.17%) | Spontaneous peritonitis  (1, 2.78%) |  |
|  |  | / | Unknown causes  (4, 11.11%) |  |
| Effusion types | Pleural effusion | 15 (62.5%) | 26 (72.22%) | 0.5720 |
|  | Peritoneal effusion | 9 (37.5%) | 10 (27.78%) |  |
