# Supplementary material for: A non-invasive secreted protein-based gene signature for prognostic stratification and tumor microenvironment assessment in gastric cancer
Source: PeerJ. 2026 Jan 13;14:e20517. doi: 10.7717/peerj.20517 (PMC12810363; doi:10.7717/peerj.20517)
Supplement: Supplemental Information 13 [file peerj-14-20517-s013.docx]

| **Supplementary Table 4** Clinical and pathological information of 52 GC cases available for immunohistochemical data of SERPINE1 in our center | | |
| --- | --- | --- |
| Characteristics | N=52 | |
| Age (average ± SD) | 65.31±10.25 | |
| Gender | Male | 38 (73.08%) |
|  | Female | 14 (26.92%) |
| Pathological types | Adenocarcinoma | 45 (86.54%) |
|  | Neuroendocrine  carcinoma | 1 (1.92%) |
|  | Adenoneuroendocrine  carcinoma | 6 (11.54%) |
| Grade | Well/moderate | 19 (36.54%) |
|  | Poor | 33 (63.46%) |
| T-stage | T1 | 8 (15.38%) |
|  | T2 | 4 (7.69%) |
|  | T3 | 14 (26.92%) |
|  | T4 | 26 (50%) |
| N-stage | N0 | 15 (28.85%) |
|  | N1 | 12 (23.08%) |
|  | N2 | 8 (15.38%) |
|  | N3 | 17 (32.69%) |
| M-stage | M0 | 52(100%) |
|  | M1 | 0 |
| Tumor Stage | Ⅰ | 7 (13.46%) |
|  | Ⅱ | 17 (32.46%) |
|  | Ⅲ | 28 (53.85%) |
|  | Ⅳ | 0 |
