# Supplementary material for: A non-invasive secreted protein-based gene signature for prognostic stratification and tumor microenvironment assessment in gastric cancer
Source: PeerJ. 2026 Jan 13;14:e20517. doi: 10.7717/peerj.20517 (PMC12810363; doi:10.7717/peerj.20517)
Supplement: Supplemental Information 14 [file peerj-14-20517-s014.docx]

| **Supplementary Table 5** Clinical and pathological information of 47 GC cases available for immunohistochemical data of α-SMA in our center | | |
| --- | --- | --- |
| Characteristics | N=47 | |
| Age (average ± SD) | 66.77 ± 10.47 | |
| Gender | Male | 38 (80.85%) |
|  | Female | 9 (19.15%) |
| Pathological types | Adenocarcinoma | 40 (85.10%) |
|  | Neuroendocrine carcinoma | 1 (2.13%) |
|  | Adenoneuroendocrine carcinoma | 6 (12.77%) |
| Differentiation grade | Well/moderate | 18 (38.30%) |
|  | Poor | 29 (61.70%) |
| T-stage | T1 | 6 (12.778%) |
|  | T2 | 2 (4.26%) |
|  | T3 | 12 (25.53%) |
|  | T4 | 27 (57.45%) |
| N-stage | N0 | 13 (27.66%) |
|  | N1 | 11 (23.40%) |
|  | N2 | 8 (17.02%) |
|  | N3 | 15 (31.91%) |
| M-stage | M0 | 47(100%) |
|  | M1 | 0 |
| Tumor Stage | Ⅰ | 6 (12.77%) |
|  | Ⅱ | 13 (27.66%) |
|  | Ⅲ | 28 (59.57%) |
|  | Ⅳ | 0 |
